# The scramblases VMP1 and TMEM41B are required for primitive endoderm specification by targeting WNT signaling

**DOI:** 10.1038/s41418-024-01435-x

**Published:** 2024-12-18

**Authors:** Markus Holzner, Tea Sonicki, Hugo Hunn, Federico Uliana, Weijun Jiang, Vamshidhar R. Gade, Karsten Weis, Anton Wutz, Giulio Di Minin

**Affiliations:** 1 Institute of Molecular Health Sciences, Department of Biology, ETH Zurich, Zurich, Switzerland; 2 Institute of Biochemistry, Department of Biology, ETH Zurich, Zurich, Switzerland; 3 Present Address: Johannes Gutenberg University of Mainz, Mainz, Germany

**Keywords:** Proteomics, Autophagy, Development, Gene expression

## Abstract

The ER-resident proteins VMP1 and TMEM41B share a conserved DedA domain, which confers lipid scramblase activity. Loss of either gene results in embryonic lethality in mice and defects in autophagy and lipid droplet metabolism. To investigate their role in pluripotency and lineage specification, we generated Vmp1 and Tmem41b mutations in mouse embryonic stem cells (ESCs). We observed that ESCs carrying mutations in Vmp1 and Tmem41b show robust self-renewal and an unperturbed pluripotent expression profile but accumulate LC3-positive autophagosomes and lipid droplets consistent with defects in autophagy and lipid metabolism. ESCs carrying combined mutations in Vmp1 and Tmem41b can differentiate into a wide range of embryonic cell types. However, differentiation into primitive endoderm-like cells in culture is impaired, and the establishment of extra-embryonic endoderm stem (XEN) cells is delayed. Mechanistically, we show the deregulation of genes that are associated with WNT signaling. This is further confirmed by cell surface proteome profiling, which identified a significant reduction of the WNT-receptor FZD2 at the plasma membrane in Vmp1 and Tmem41b double mutant ESCs. Importantly, we show that transgenic expression of Fzd2 rescues XEN differentiation. Our findings identify the role of the lipid scramblases VMP1 and TMEM41B in WNT signaling during extra-embryonic endoderm development and characterize their distinct and overlapping functions.

## Introduction

Vesicle trafficking and membrane synthesis are intricately interconnected within cellular signaling networks. Lipid scramblases, with their property to shape lipid membrane composition, play a crucial role in facilitating inter-organelle communication. Beyond their integral involvement in maintaining cell homeostasis, recent studies have unveiled their implications in various pathological conditions, such as viral infection [1–5], cancer development [6–11], and inflammatory diseases [12–16]. The vacuole membrane protein 1 (VMP1) and transmembrane protein 41B (TMEM41B) are lipid scramblases with a highly conserved DedA domain [17, 18]. They localize at the ER membrane and are distinguished from other ER-associated flippases or scramblases as they facilitate bi-directional lipid diffusion in an ATP- and Ca2+-independent manner. VMP1 and TMEM41B are necessary for autophagosome formation [19, 20], lipid droplet metabolism [17, 21], and lipoprotein formation [22]. Additional reports proposed a more global role in ER protein homeostasis [23] and vesicle transport [24]. Despite multifaceted functions, their absence does not influence cell survival significantly. Compensatory mechanisms shared by VMP1 and TMEM41B or other ER scramblases likely maintain cellular homeostasis. While VMP1 depletion leads to a pronounced defect in autophagosome assembly [19, 20], TMEM41B appears to play a pivotal role in sustaining lipid droplet homeostasis and serves as a crucial factor in RNA virus replication [3, 25]. Differences in cellular systems and depletion methods across studies complicate comparisons of VMP1 and TMEM41B functions. While their role in autophagy has been directly compared [26], similar studies are needed to assess their specificity or redundancy in other cellular functions.

VMP1 and TMEM41B are both essential during embryonic development. Vmp1 [21] and Tmem41b [27] deficient mice die around E8.5 with a severe developmental delay. Previously, defects in the visceral endoderm function have been proposed. In particular, loss of Vmp1 has been suggested to block the synthesis and release of lipoproteins from the visceral endoderm. However, mice with defects in lipoprotein biogenesis experience mortality at a later stage, E10.5 [28, 29]. This observation suggests a distinct and earlier involvement of VMP1 and TMEM41B in embryo development.

During implantation, the mouse embryo is composed of three lineages: the trophectoderm (TE), the primitive endoderm (PE), and the epiblast (EPI), which develops into the embryo [30]. The EPI and PE are specified within the inner cell mass (ICM) of the blastocyst. Defects in the formation of these lineages are challenging to characterize in vivo as they appear pre-implantation and due to the small embryo size. To investigate the role of Vmp1 and Tmem41b in early embryonic lineage specification, we generated mutations in Vmp1 and Tmem41b in ESCs. We show that a combined Vmp1 and Tmem41b mutation in ESCs results in a specific defect in PE formation and a delayed conversion to extra-embryonic endoderm cells (XEN). This delay is caused by reduced WNT signaling and can be rescued through WNT activation.

## Results

### Vmp1 and Tmem41b mutant ESCs accumulate LC3-positive autophagosomes and lipid droplets

**Fig. 1:**
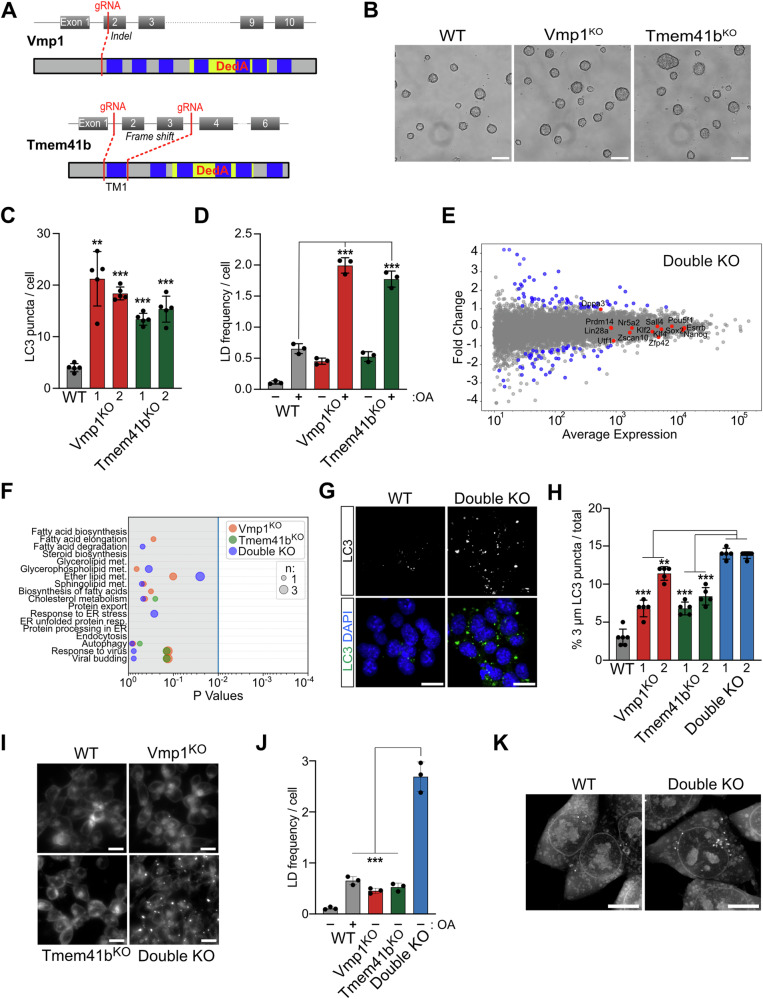
Vmp1 and Tmem41b double KO ESCs display increased autophagosome and lipid droplet accumulation. **A** Schematic representation of the CRISPR/Cas9-mediated KO strategy for Vmp1 and Tmem41b genes. For depleting Vmp1 (top), a single gRNA inducing an indel mutation has been used. In the case of the Tmem41b gene (bottom), two gRNAs were used to delete exons 2 and 3. The sequences coding for transmembrane domains (TM) are indicated in blue, the conserved DedA domain is highlighted in yellow, red lines indicate cut sites of gRNAs. **B** Colony morphology of VMP1^KO^ and Tmem41^KO^ ESCs remains unchanged compared to WT ESCs. Scale bar: 100 µm. **C** VMP1 and Tmem41b depletion induce accumulation of autophagosomes in ESCs. The graph shows the quantification of LC3 clusters per cell of a representative experiment (see Fig. S2A). Data were collected from more than 200 cells. For each KO condition, two independent clones have been analyzed. Dots correspond to the frames analyzed per sample. Data are represented as mean ± SD. The P-value is relative to the WT sample. **D** Vmp1 and Tmem41b depletion leads to the accumulation of lipid droplets (LD) in ESCs. Graph quantifies LD per cell in WT and KO ESCs (see Fig. S2D). ± oleic acid treatment. At least 500 cells were analyzed for every condition; dots represent independent biological replicates. Data are represented as mean ± SD. **E**, **F** Transcriptional analysis of Double KO ESCs did not identify many deregulated genes. **E** Shown are differentially regulated genes in Double KO ESCS in blue as MA plot. Genes highlighted in red are pluripotency markers. **F** Differentially regulated genes do not cluster in essential processes. The graph shows differentially expressed genes of single and double KO ESCs by cellular processes. Dot size correlates to the number of deregulated genes within a cellular process. **G**, **H** Double KO ESCs accumulate LC3-positive autophagosomes of increased size. **G** Shown are representative IF images of WT and double KO ESCs stained for LC3. Nuclei are stained with Dapi. Scale bar: 20 µm. **H** Plotted is the quantification of LC3 clusters of 3 µm in diameter. For each condition, two independent clones have been analyzed. Dots correspond to the frames analyzed per sample. Data were collected from more than 200 cells. Data are represented as mean ± SD. The *P*-value is relative to the Double KO sample with the highest SD. (**I-J**) Double KO ESCs accumulate LDs without oleic acid treatment. **I** Live cell staining of WT, Vmp1, Tmem41b, and double KO ESCs with BODIPY. Scale bar: 20 µm. **J** The number of LDs per cell is plotted for all KO clones. Data are represented as mean ± SD. **K** Double KO ESCs present an increased amount of LD visualized through refractive index microscopy. Scale bar: 10 µm.

To assess the influence of a Vmp1 or Tmem41b depletion on mouse ESCs and in early embryo development, we engineered genomic deletions using CRISPR/Cas9 targeted mutagenesis. Vmp1 was mutated by introducing a frameshift in exon 2 (Fig. 1A). Loss of VMP1 protein was confirmed in independent mutant ESC clones (Vmp1^KO^) (Fig. S1A). To abrogate Tmem41b expression, we deleted the genomic region between exons 2 and 3 (Fig. 1A), which encodes the first transmembrane domain of the protein (Tmem41b^KO^). This mutation is predicted to compromise the protein topology. Additionally, the splicing of exon 1 to exon 4 produces a frameshift, suggesting a null allele. The deletion was confirmed on the genomic DNA (Fig. S1B). Tmem41b mRNA levels were strongly reduced (Fig. S1C, D).

Vmp1 and Tmem41b mutant ESCs showed similar morphology (Fig. 1B), proliferation (Fig. S1E), and expression of pluripotency markers (Fig. S1F, G) compared to WT ESCs. We confirmed that the pluripotency network was unchanged by a transcriptomic analysis of Vmp1 and Tmem41b mutant cells. This analysis also showed that the transcription profile of cells was marginally affected, consistent with earlier results of ESC characterization (Fig. S1I, J and Table S2).

Considering the role of Vmp1 and Tmem41b in autophagy, we assessed the accumulation of LC3-positive autophagosomes by microscopy to determine the impact of their mutations. We found an increase of LC3 clusters in both mutants compared to WT ESCs (Figs. 1C and S2A, B). Interestingly, a similar increase of LC3 clusters was observed in Vmp1 (4 times) and Tmem41b (3,5 times) mutant cells. Next, mutant cells were analyzed for the retention of lipid droplets (LD). To amplify LDs, we treated all samples with oleic acid (OA) and visualized LDs by BODIPY staining. We observed that Vmp1 and Tmem41b mutations led to a 4-fold increase of LD numbers with respect to WT cells (Figs. 1D and S2D, E). These results suggest that both Vmp1 and Tmem41b contribute to autophagosome and lipid droplet metabolism in ESCs, recapitulating earlier studies performed in other cellular systems [17, 20]. The direct comparison of both mutations revealed a similar contribution of Vmp1 and Tmem41b to both processes.

### Vmp1 and Tmem41b activity is not required for ESC self-renewal

We generated double mutant ESC lines to characterize potential redundant functions between VMP1 and TMEM41B. A Tmem41b mutation was engineered in Vmp1 mutant ESCs (Double KO) (Fig. S1A–D). Several independent mutant clones were obtained. Notably, Double KO cells showed a regular ESC morphology (Fig. S1H) and proliferation rate (Fig. S1E). The absence of Vmp1 and Tmem41b did not affect secretory compartments, as ER, Golgi apparatus, and late endosomes appeared normal in mutant cells (Fig. S3A). Transcriptomics analysis of Double KO cells showed a limited perturbation in gene expression (Fig. 1E). The combined mutations did not result in a greater number of differentially regulated genes compared to Vmp1^KO^ cells (Fig. S3C). Minimal overlap of differentially expressed genes was detected among Vmp1, Tmem41b, and Double KO mutant cell lines (Fig. S3B, C). Additionally, we did not observe enrichment for genes associated with defined cellular processes (Figs. 1F and S3D, E). The pluripotency network, as also shown by IF staining (Fig. S1F, G), was not perturbed in Double KO cells. We conclude that Vmp1 and Tmem41b are not essential for ESC self-renewal.

### Vmp1 and Tmem41b have redundant functions in autophagosome and lipid droplet formations

LC3 staining showed that Double KO cell lines present an exacerbated phenotype in autophagosome accumulation compared to single mutants. Although the total number of LC3-positive autophagosomes per cell remained unchanged (Figs. 1G and  S2A, B), we observed an increased size. 14% of LC3 clusters in the Double KO had a larger size (>3 μm), compared to the 8% of autophagosomes across the single mutations and 3% in the WT condition (Figs. 1H and S2C).

The simultaneous depletion of Vmp1 and Tmem41b also had a greater impact on LD formation. Double KO cells showed a drastic increase in LD number in the absence of oleic acid treatment, whereas in WT and Vmp1 and Tmem41b mutant ESCs, LDs became detectable after OA treatment (Figs. 1I, J and S2D, E). As BODIPY could also stain other lipid-containing compartments [31], we used the labeling-independent technique of refractive index tomography to visualize the lipid droplets [32, 33] and confirmed the enrichment of lipid droplets in the Double KO cells (Figs. 1K and S2F, G). Together, these results indicate that Vmp1 and Tmem41b have overlapping functions in ESCs, as the combined mutation of both genes exacerbates autophagosome and lipid droplet accumulation.

### Loss of Vmp1 and Tmem41b affects ESC potential to differentiate into primitive endoderm

**Fig. 2:**
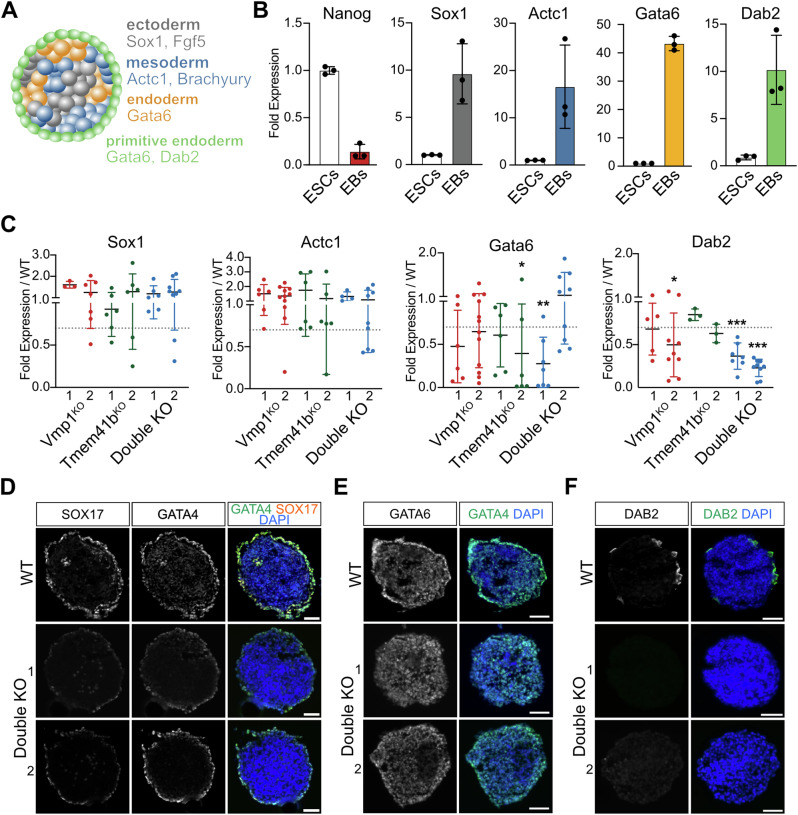
Double KO ESCs specify all germ layers, but not the primitive endoderm. **A** Schematic representation of an Embryonic Body (EB) which consists of 3 different lineages. The ectoderm expresses Fgf5 and Sox1 (gray), the mesoderm Actc1 and Brachyury (blue), and the endoderm by Gata6. The surrounding region of the EB corresponds to the primitive endoderm (green) and is marked by Gata6 and Dab2 expression. **B** ESCs differentiated to EBs express lineage markers for ectoderm (Fgf5), mesoderm (Brachyury), endoderm (Gata6), and primitive endoderm (Dab2). Graphs show mRNA levels of WT EBs normalized to the Sdha gene. Dots represent biological independent experiments. Data are represented as mean ± SD. **C** Double KO ESCs specifically show defects in expressing the primitive endoderm marker, Dab2. qPCR analysis for Sox1, Actc1, Gata6, and Dab2 mRNA expression in mutant EBs relative to WT EBs. Two independent clones were analyzed per cell line. Each dot represents an independent experiment. Data are represented as mean ± SD. The line at 0,7 indicates an arbitrary threshold after which successful expression of the marker is considered. Statistical significance (Welch’s *t*-test) is denoted as follows: ns: *p* > 0.05, *p < 0.05, **p < 0.01, ***p < 0.001. Double mutant EBs show decreased expression of endoderm markers. Immunofluorescence images of EB sections stained for the endoderm markers GATA4 and SOX17 (**D**), GATA6 (**E**), DAB2 (**F**). WT in the top panel, two independent clones for Double KO condition in the center and bottom panels. Nuclei are stained with Dapi. Scale bar: 50 µm.

To evaluate the effect of Vmp1 and Tmem41b on the differentiation potential of ESCs, we used embryoid body (EB) differentiation (Fig. 2A). After 6 days of differentiation, EBs derived from WT ESCs showed increased expression of ectoderm (Sox1 and Fgf5), mesoderm (Actc1 and Brachyury) and endoderm (Gata6 and Dab2) genes (Figs. 2B and S4A), while the expression of pluripotency markers (Nanog and Pou5f1) was lost (Figs. 2B and S4A). We evaluated variations in the differentiation potential of mutant cells by comparing the expression of these markers by RT-qPCR. For each mutant, the differentiation potential of two independent clones was tested. Vmp1, Tmem41b, and double mutant ESCs formed well-defined EBs that resembled those of WT cells. All cell lines efficiently differentiated into the ectoderm and mesoderm lineages (Figs. 2C and S4B, C). Notably, Double KO EBs showed a decreased expression of the endoderm markers Dab2 (100%) and Gata6 (57% of experiments) (Figs. 2C and S4C). Neither single mutant showed a similar failure in Dab2 expression, even if some variability was detected between the analyzed clones. In the single mutants, we noticed a decreased endoderm specification, as shown by Gata6 expression levels in experiments performed with Vmp1^KO^ (56% of experiments) and Tmem41b^KO^ (58%) cells (Figs. 2C and S4C). Our results indicate that combined mutation of Vmp1 and Tmem41b leads to reduced Dab2 expression and predisposes to variability in the expression of other endodermal genes.

We confirmed the differentiation potential of Double KO cells into ectodermal lineages by generating SOX1-positive spinal cord organoids (Fig. S4D). Mesodermal differentiation was inferred from the ability of double mutant EBs to form contractile cardiomyocytes after plating (Video S1–2). We subsequently sectioned day 6 EBs to evaluate protein expression and distribution of endoderm markers by IF. Expression of GATA4, GATA6, and SOX17 was detected in all conditions but noticeably decreased in Double KO compared to WT EBs (Fig. 2D, E). Interestingly, decreased GATA4, GATA6, and SOX17 staining was pronounced in EB border regions, which resemble the primitive endoderm layer [34]. In line with these findings, we confirmed the absence of DAB2 in Double KO EBs (Fig. 2F). DAB2 is specifically expressed in the primitive endoderm, while GATA4 and GATA6 are known to be also expressed in definitive endoderm.

The significant reduction in both mRNA and protein levels of Dab2, along with the weakened expression of endoderm markers at the edges of embryoid bodies, provides evidence that the absence of Vmp1 and Tmem41b compromises the formation of primitive endoderm.

### Combined Vmp1 and Tmem41b mutations delay ESC differentiation into extra-embryonic endoderm stem (XEN) cells

**Fig. 3:**
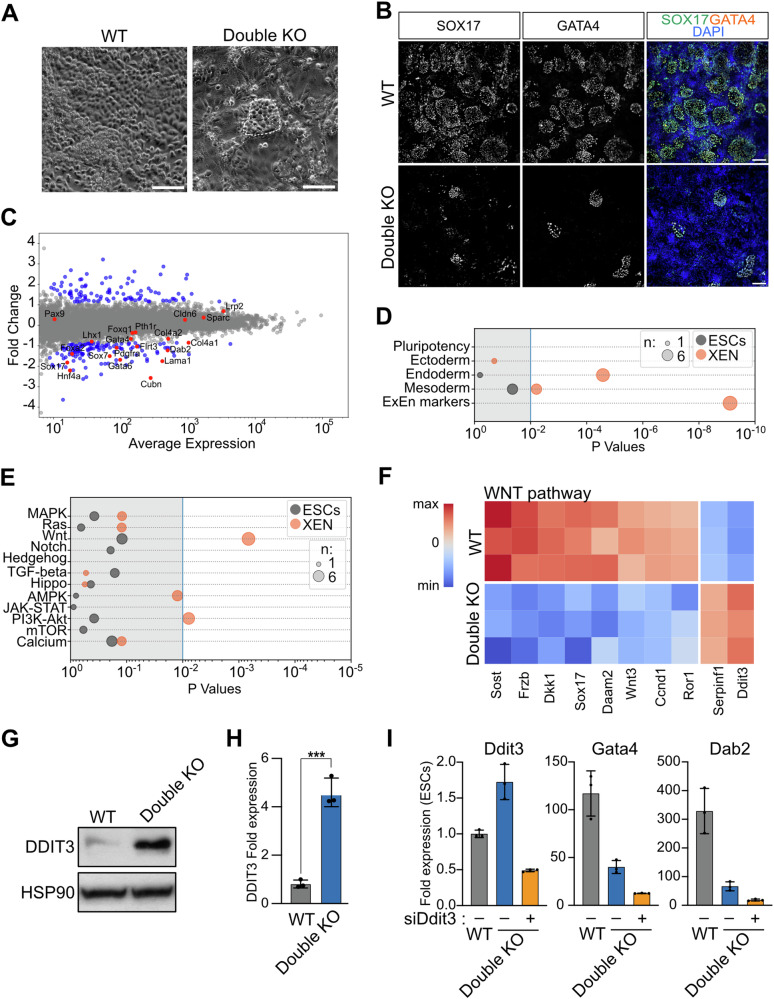
XEN differentiation in Double KO cells is delayed. **A**, **B** Double KO ESCs show a delay in XEN differentiation. **A** Brightfield images of XEN cells at day 6 of differentiation in WT and Double KO cells. XEN islands are circled in white in the Double KO condition. Scale bar: 100 µm. **B** Immunofluorescence images of day 6 XEN cells stained for SOX17 and GATA4. Nuclei are stained with Dapi, Scale bar: 150 µm. **C**–**F** Transcriptional analysis of XEN cells. **C** Differentially expressed genes between Double KO and WT cells at day 2 during XEN specification. XEN markers are labeled in red; differentially expressed genes in blue. **D**, **E** GO-Term analysis of differentially expressed genes between Double KO and WT conditions in ESCs and XEN cells in the context of developmental processes (**D**) and signaling pathways (**E**). Dot size corresponds to the number of differentially regulated genes. (**F**) WNT pathway-related genes differentially expressed in Double KO cells. Heatmap shows min to max expression of the average of three biological replicates for WT and Double KO XEN cells. **G**, **H** VMP1 and Tmem41b depletion promotes the increase of DDIT3 protein levels. **G** Western Blot analysis of DDIT3 expression in WT and Double KO cells differentiated to XEN cells for 2 days. HSP90 is shown as loading control. **H** Quantification of increased DDIT3 protein levels in three biological replicates. Expression relative to WT XEN cell and normalized to respective HSP90 expression. Data are represented as mean ± SD. **I** DDIT3 silencing does not restore XEN specification in Double KO cells. qPCR analysis at day 3 of differentiation for Ddit3, Gata4, and Dab2 mRNA expression in mutant cells relative to WT ESCs transfected without and with Ddit3 siRNA. Data are represented as mean ± SD (*n* = 3).

We tested the ability of Double KO ESCs to differentiate into extra-embryonic endoderm stem (XEN) cells to characterize endodermal differentiation further. XEN cells share characteristics with PE cells including cell signaling, gene expression, and differentiation potential [35]. Double KO and WT cells were differentiated into XEN cells with Activin A and Retinoic Acid [36]. After 6 days, WT ESCs efficiently differentiated into XEN clusters (Fig. 3A), exhibiting a stellate and refractile morphology [36]. In Double KO cells, the formation of XEN clusters was substantially reduced. We characterized XEN identity by analyzing the expression of XEN markers by RT-qPCR (Fig. S5A) and immunostaining (Figs. 3B and S5B). It is worth noting that the small number of derived XEN cells from Double KO ESCs can be expanded for multiple passages, as shown in Fig. S5C. This result suggests that the loss of Vmp1 and Tmem41b does not entirely prevent XEN specification but rather causes a delay and inefficiency in the differentiation process.

### WNT signaling is decreased in Vmp1 and Tmem41b mutant cells during XEN specification

To characterize the effect of the Vmp1 and Tmem41b double mutation on XEN specification, we analyzed the transcriptomic profile of Double KO and WT cells during the differentiation process (Table S3). On day two of differentiation, we detected the induction of XEN-associated genes [37] in WT cells. However, in Double KO cells these genes were weakly expressed (Figs. 3C, D and S5E). Intriguingly, we did not observe any perturbation in the transcription of genes involved in lipid metabolism, autophagy, or ER maintenance (Fig. S5D).

Instead, Double KO cells show consistent deregulation of genes associated with the WNT pathway (Fig. 3E, F). A close analysis of these genes allowed to identify several well-established WNT-target genes, including Dkk, Frzb, Sox17, Wnt3, and Ccnd1 [38–41]. Their down-regulation strongly suggested a decreased activity of the WNT pathway in Double KO cells and might explain the observed defect in XEN specification [42]. Interestingly, Ddit3 was strongly upregulated in Double KO cells. DDIT3 acts as an inhibitor of the WNT signaling by repressing the transcriptional activity of the TCF7/TCF4 complex [43]. We confirmed increased levels of the DDIT3 protein in Double KO cells by Western analysis (Fig. 3G) and quantified a fourfold increase over WT cells (Fig. 3H). Considering that Ddit3 is not reported to be a WNT-targeted gene, but instead, its expression is controlled by VMP1 levels [44], we verified if DDIT3 increase might explain WNT-target gene repression in mutant cells. For this purpose, we depleted Ddit3 in WT and mutant cells by RNA interference. After two days of differentiation, the transcription of XEN-specific genes was evaluated by qPCR. Despite efficient siRNA-mediated reduction of Didt3 mRNA levels (Fig. 3I), we did not observe an increase in the expression of XEN marker genes in Double KO cells.

These results indicate that the increased level of DDIT3 in mutant cells is not the only cause of the XEN specification defect caused by Vmp1 and Tmem41b mutations.

### Vmp1 and Tmem41b mutations reduce FZD2 abundance at the plasma membrane

**Fig. 4:**
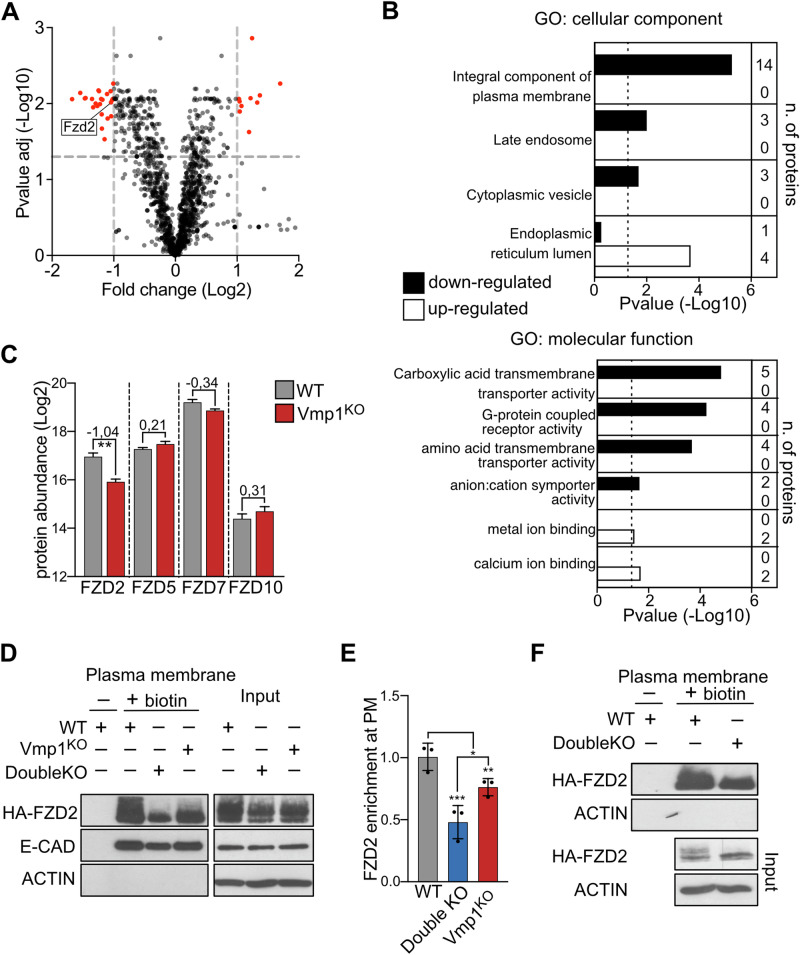
Vmp1 and Tmem41b mutant ESCs show reduced plasma membrane abundance of the WNT-receptor FZD2. **A** Differences in the abundance of plasma membrane proteins in WT and VMP1^KO^ cells. Volcano plot displaying differentially expressed proteins at the plasma membrane in WT and VMP1^KO^ ESCs. Proteins with the most significant divergence are highlighted in red. Gray lines denote the selection criteria: an absolute log2 fold-change > 1 and a false discovery rate <0.05. The position of FZD2 is annotated. **B** GO-term analysis of proteins differentially abundant at the plasma membrane of WT and Vmp1^KO^ cells. Shown are GO terms related to protein localization and molecular function. **C** Abundance of Frizzled receptors identified by MS analysis in WT and mutant cells. The average and standard error is reported for three independent biological replicates **D** Western blot analysis of HA-FZD2 abundance at the PM in WT, VMP1^KO^, and Double KO ESCs. E-CADHERIN and ACTIN serve as controls for PM purification and loading, respectively. **E** Quantification of HA-FZD2 protein levels at the PM across three biological replicates relative to WT cells and normalized to the total HA-FZD2 (Input). Data are represented as mean ± SD. **F** Western blot analysis of HA-FZD2 abundance at the PM in WT and Double KO cells differentiated into XEN cells for 2 days.

To identify potentially affected factors of cell signaling, we investigated the influence of the Vmp1 mutation on proteins at the plasma membrane (PM) in ESCs. For this purpose, we labeled and purified surface proteins in mutant and WT cells using aminooxy-biotinylation and characterized their abundance by TMT mass spectrometry (Fig. S6A, B). Our analysis revealed that a limited subset of proteins showed differential abundance at the plasma membrane in mutant cells, with 26 proteins significantly downregulated and 12 upregulated (out of 1417 detected proteins) (Fig. 4A and Table S4). This finding aligns with our RNA expression profiles, indicating that Vmp1 and Tmem41b mutations have a limited effect on the protein levels of ESCs. It also supports the conclusion that, despite defects in autophagy and lipid droplet formation, the secretory pathway remains largely intact in the absence of Vmp1. GO-term analysis highlighted that proteins more abundantly present at the PM in Vmp1 mutant cells are characterized by an ER-Golgi localization, suggesting partial defects in retrograde trafficking (Fig. 4B). Moreover, we noticed that the abundance of four GPCRs was reduced at the plasma membrane of mutant cells (Fig. 4B). Among them, we found the WNT-receptor FZD2 (Fig. 4A and Table S4). Other Frizzled receptors—FZD5, FZD7, and FZD10— were detected at the PM but remained unaffected (Figs. 4C and S6C). The decrease of FZD2 protein abundance at the PM was independent of Fzd2 transcription, as no difference in Fzd2 mRNA was detected between WT and mutant cells (Fig. S6D).

To confirm these findings and assess whether FZD2 reduction contributes to the XEN specification defects in Double KO cells, we generated ESCs with constitutive expression of HA-Fzd2 across Vmp1^KO^, Tmem41b^KO^, Double KO, and WT genotypes. We evaluated HA-FZD2 translocation to the plasma membrane using a cell-impermeable biotinylation reagent followed by purification of biotinylated proteins (Figs. 4D and S6E, F). We confirmed that FZD2 is less abundant at the plasma membrane in Vmp1 mutant cells, validating previous MS results (Fig. 4D, E). Instead, the depletion of TMEM41B had a limited effect on FZD2 amount at the plasma membrane (Fig. S6E, F). Notably, Double KO cells exhibited an even greater reduction in FZD2 levels at the PM than the one verified in Vmp1 mutant cells, suggesting an additive effect of the Vmp1 and Tmem41b mutations (Fig. 4D, E). Similar results were observed when analyzing HA-FZD2 enrichment at the plasma membrane in Double KO and WT cells during XEN specification (Fig. 4F). In this experiment, we noticed a marked reduction of the upper form of the FZD2 doublets in mutant cells. This suggests that FZD2 is subjected to different post-translational modifications in WT and mutant cells.

### FZD2 overexpression rescues XEN differentiation defects in Vmp1 and Tmem41b mutant cells

**Fig. 5:**
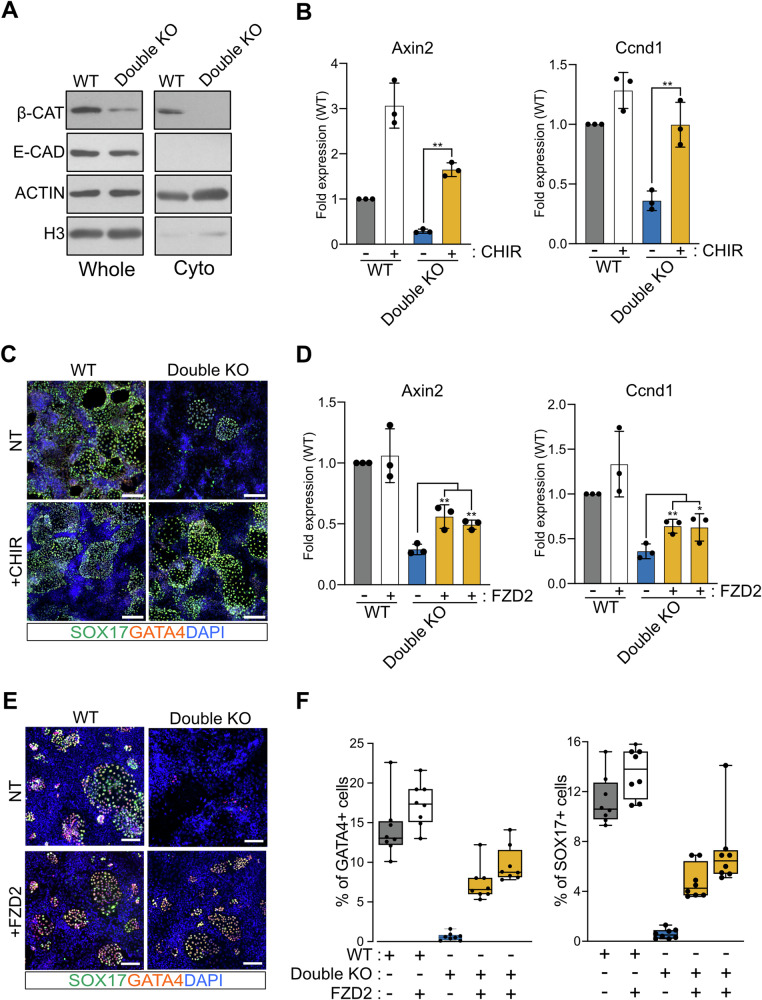
Activation of WNT signaling rescues the delay of Double KO cells in XEN differentiation. **A** Cytoplasmic β-CATENIN levels are reduced in Double KO cells. Western blot analysis was performed on whole-cell lysates (1/50) and purified cytoplasmic fractions (1/50) from WT and Double KO cells. E-CADHERIN, Histone H3, and ACTIN were used as controls for purification and loading. Whole-cell and purified cytoplasmic fractions are shown with the same exposure conditions. **B**, **C** Chiron-dependent WNT activation rescues the delay of Double KO XEN differentiation. **B** Expression of WNT-target genes in WT and Double KO cells upon CHIR treatment. Graphs show relative Axin2 and Ccnd1 mRNA levels normalized to the Gapdh gene. Dots represent biological independent experiments. Data are represented as mean ± SD. **C** IF staining for SOX17 and GATA4; nuclei are stained with Dapi. Scale Bar: 150 µm. **D**–**F** FZD2 overexpression rescues the differentiation delay in Double KO XEN cells. **D** Expression of WNT-target genes in WT and Double KO cells overexpressing FZD2. Graphs show relative Axin2 and Ccnd1 mRNA levels normalized to the Gapdh gene. Dots represent biological independent experiments. Data are represented as mean ± SD. **E** Immunofluorescence staining for SOX17 and GATA4 in WT and Double KO cells. Nuclei are counterstained with DAPI. Scale bar: 100 µm. **F** Quantification of GATA4 and SOX17 positive cells during XEN differentiation, expressed as a percentage of total cells (DAPI-stained).

Our observation of deregulated WNT-target genes and a reduced abundance of FZD2 at the PM indicated that WNT signaling defects might impair XEN specification. To investigate if indeed the WNT signaling cascade is affected, we quantified β-CATENIN, a downstream protein and major signal transducer of the WNT pathway. We observed a marked decrease of β-CATENIN protein in Double KO compared to WT cells (Fig. 5A). This difference was even stronger, specifically analyzing the cytoplasmic fraction of β-CATENIN that is not associated with cadherins. To explore if the defect in XEN differentiation of double mutant cells can be rescued by chemically activating WNT signaling, we employed the GSK3 kinase inhibitor (CHIR) to prevent β-CATENIN degradation. We confirmed that CHIR treatment increases β-CATENIN levels in ESCs (Fig. S7A) and promotes the expression of WNT-target genes during XEN specification (Figs. 5B and S7B). Notably, we verified that in Double KO cells, the expression of these markers was decreased compared to WT cells, and GSK3 inhibition was sufficient to restore their levels (Figs. 5B and S7B). CHIR had no additional effect on XEN specification in WT ESCs. However, GSK3 inhibition significantly enhanced XEN cell cluster formation in double mutant ESCs (Figs. 5C and S7C, D). Finally, to investigate whether also overexpression of FZD2 would rescue XEN differentiation defects in Double KO cells, we generated subclones with high FZD2 expression for both Double KO and WT ESCs (Fig. S8A). The increased amount of FZD2 alone did not further stimulate the WNT pathway or XEN specification in WT cells (Figs. 5D–F and S8B–D). However, FZD2 overexpression in Double KO cells sustained the expression of WNT-target genes (Figs. 5D and S8B) and was sufficient to restore mutant cell potential for differentiation into XEN cells (Figs. 5E, F and S8C, D). Collectively, our data implicate Vmp1 and Tmem41b in WNT signaling during primitive endoderm specification.

### Vmp1 and Tmem41b depletion prevents correct maturation and secretion of the FZD2 receptor

**Fig. 6:**
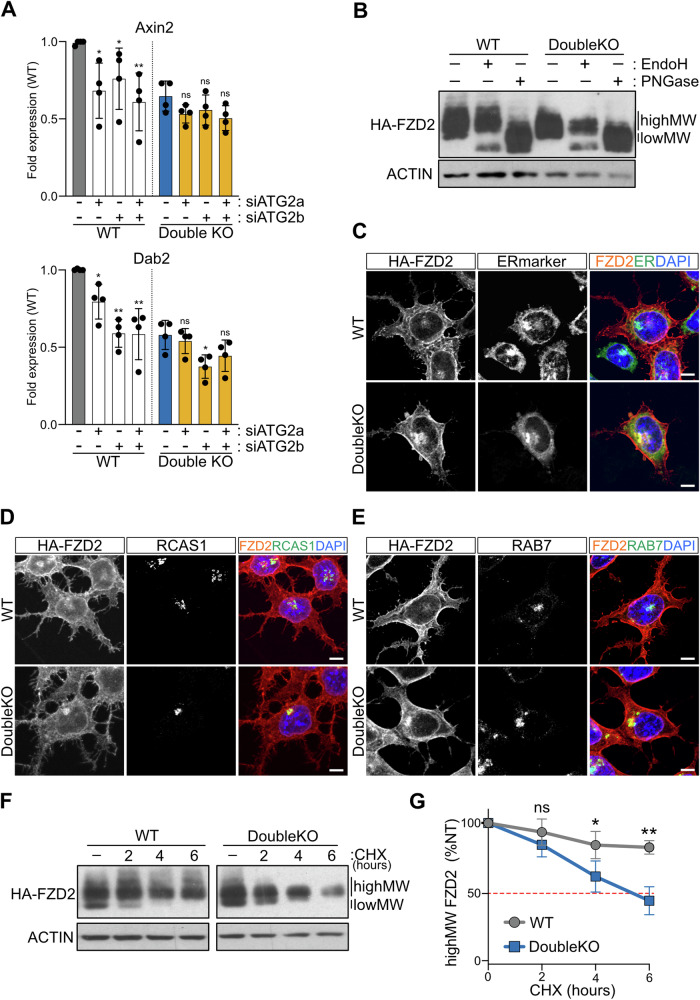
FZD2 maturation is impaired in Double KO cells. **A** Silencing of Atg2 mimics the effect of the depletion of Vmp1 and Tmem41b. Expression of WNT-target genes and XEN markers in WT and Double KO cells upon Atg2 down-regulation. Graphs show relative Axin2 and Dab2 mRNA levels normalized to the Gapdh gene. Dots represent biological independent experiments. Data are represented as mean ± SD. **B** Glycosylation pattern of FZD2 in WT and Double KO cells. Western blot analysis on lysates treated with EndoH and PNGase recombinant proteins. Bands corresponding to the low (lowMW) and high (highMW) FZD2 forms before enzymatic treatment are indicated. ACTIN is shown as loading control. Localization of HA-FZD2 in WT and Double KO cells during XEN specification at the ER (ERmarker) (**C**), Golgi compartment (RCAS1) (**D**), and late endosomes (RAB7) (**E**). Nuclei are counterstained with DAPI. Scale bar: 5 µm. **F**, **G** Stability of the high molecular forms of the FZD2 receptor is decreased in Double KO cells. **F** Western blot analysis of WT and Double KO cells differentiated into XEN lineages for two days and treated with the protein synthesis inhibitor CHX (20 μg/ml) for the specified times. Bands corresponding to the low (lowMW) and high (highMW) FZD2 forms are indicated. ACTIN is shown as loading control.

In Double KO cells, defects in protein trafficking may arise from changes in autophagosome and lipid droplet metabolism or result from ER stress triggered by the lack of these scramblases. ATG2 interacts with both VMP1 and TMEM41B [18], playing a vital role in the transport of lipids to autophagosomes and lipid droplets. In mice, two genes encoding highly homologous ATG2 proteins have been described. To investigate the potential role of lipids in controlling FZD2 secretion, we downregulated Atg2a and Atg2b expression in ESCs by RNA interference and analyzed the effect of their depletion during XEN specification. The knockdown of Atg2 genes significantly reduced the expression of WNT-target genes and XEN markers in WT cells (Figs. 6A and S9A). In contrast, this effect was either absent or markedly reduced in Double KO cells, supporting the idea that Vmp1/Tmem41b and Atg2 function in an interdependent manner. The observation that Atg2 depletion mimics the effect of VMP1 and TMEM41B loss during XEN specification suggests the requirement of a functional lipid transport machinery for effective WNT pathway activation.

To investigate how the scramblase activity of VMP1 and TMEM41B might impact FZD2 levels at the plasma membrane (PM), we characterized the different forms of FZD2 protein in WT and mutant cells (Fig. 4F). GPCRs are frequently subjected to glycosylation, a modification essential for proper receptor folding, secretion, and activity [45]. The distinct molecular forms of the HA-FZD2 protein detected by SDS-PAGE represent varying glycosylation levels on the receptor (Fig. 6B). The lower molecular band (lowMW) is sensitive to EndoH treatment, indicating basic N-glycosylation, which typically occurs in the ER. In contrast, the higher molecular weight forms (highMW) are sensitive to PNGase but unaffected by EndoH, corresponding to complex glycans added in the Golgi, and marking the mature receptor. Our analysis revealed a significant reduction in the fraction of FZD2 with complex glycans in Double KO cells (Fig. 6B). Analyzing protein localization by immunofluorescence, we observed that FZD2 displays a dispersed pattern in WT cells, likely reflecting proper receptor translocation to the plasma membrane (PM) and endocytic compartments (Fig. 6C, D). Instead, in mutant cells, we observed accumulation of the protein in ER and Golgi subdomains, suggesting retention of FZD2 forms during the maturation process. Additionally, FZD2 co-localizes with the late-endosome marker RAB7 predominantly in mutant cells (Fig. 6E).

Further analysis showed that FZD2 is less stable in cells lacking Vmp1 and Tmem41b. Following inhibition of protein synthesis, lower molecular weight FZD2 forms (lowMW) reduced rapidly and similarly in both WT and mutant cells (Figs. 6F and S9B). Instead, FZD2 fractions with complex glycans (highMW) remained highly stable in WT cells but declined rapidly in Double KO cells (Fig. 6G). These results suggest that in the absence of VMP1 and TMEM41B, FZD2 stabilization at the PM is impaired, likely due to defects in the receptor maturation process.

## Discussion

Vmp1 and Tmem41b have recently been identified in a number of genetic screens designed to study various cellular processes, including autophagy, lipid metabolism, virus maturation, and ER stress [22]. In this study, we have unveiled the role of Vmp1 and Tmem41b in regulating protein secretion. Our findings indicate that their presence is essential for the trafficking of the WNT-receptor FZD2 to the plasma membrane. A drastic reduction of FZD2 in mutant cells impairs WNT signals and causes an XEN cell differentiation defect. Our data, therefore, implicate a function of Vmp1/Tmem41b in WNT signaling. Given that various stimuli influence Vmp1 expression and activity [46, 47], the interaction between Vmp1 and Tmem41b within the secretory pathway is critical for physiological and pathological processes, such as during tumorigenesis.

The pleiotropic function and peculiar structure of the DedA domain found in both scramblases have prompted multiple studies addressing its mode of action and role in cellular homeostasis. An important point under evaluation is whether VMP1 and TMEM41B work cooperatively or show specialization for different cellular processes. To address this question, we targeted Vmp1 and Tmem41b genes in ESCs and introduced mutations abrogating their expression. This allowed us to directly compare the specificity of VMP1 and TMEM41B in multiple processes. We report that their absence similarly affects autophagy and lipid droplet accumulation. Moreover, we show that the simultaneous loss of both genes synergizes and exacerbates defects of single mutants, particularly in LD formation. Notably, loss of both genes did not affect self-renewal in ESCs and had only subtle effects on gene expression. Our characterization shows that Vmp1 and Tmem41b perform overlapping functions in ESCs. It will be interesting to understand if variating expression levels of Vmp1 and Tmem41b can account for their redundancy or dominance in different cellular systems.

The embryonic lethality caused by Vmp1 and Tmem41b mutations [21, 27] during development may be explained by the defective primitive endoderm specification that we detected in vitro. Embryoid body experiments and the direct differentiation of ESCs to extra-embryonic endoderm stem (XEN) cells support a specific delay in PE formation in double mutant cells. Interestingly, it has been reported that other mutations leading to PE delay cause retardation and embryo lethality at E8.5 [48], mirroring phenotypes of Vmp1 or Tmem41b mutant embryos. While both scramblases are needed in mice for normal development, the depletion of both genes is required to cause a differentiation delay in ESCs. More permissive in vitro growth conditions may explain the requirement for the depletion of both genes to manifest a measurable differentiation defect in cell culture. It is worth noting that single Vmp1^KO^ or Tmem41b^KO^ cells also showed subtle defects in endoderm specification in embryoid bodies, albeit less consistently than Double KO cells (Fig. 2C). The finding of WNT signaling deregulation in Vmp1 and Tmem41b double mutant XEN cells explains the specification delay detected in mutant cells. This is consistent with earlier reports that the WNT/TCF7L1 transcriptional response is necessary in vitro and in vivo for PE expansion [42].

Overexpression of the FZD2 receptor rescues the defects of Vmp1 and Tmem41b double mutant cells during XEN specification. We propose that the decreased availability of FZD2 at the plasma membrane in mutant cells causes a delay in WNT signaling activation and PE defects. The observation that Fzd2 mutant mice exhibit developmental defects at later stages [49] suggests the possibility of compensatory mechanisms among different Frizzled receptors in vivo. Future studies will focus on the deep characterization of other potential GPCRs subjected to Vmp1/Tmem41b regulation. While our results indicate that the increased expression of the WNT repressor DDIT3 alone does not fully account for the delay in XEN specification, it may still contribute to this delay during embryonic development. The elevated levels of DDIT3 in mutant cells might exert an additive effect, further reducing WNT pathway activity.

Vmp1 and Tmem41b emerge as key factors in the secretary machinery of cells. Beyond their established roles in lipid and membrane metabolism, we demonstrate their involvement in regulating protein trafficking at the plasma membrane. As in the case of lipid droplet accumulation, the absence of both scramblases leads to a synergetic effect in controlling FZD2 secretion. Notably, this effect is transcription independent, as evidenced by expression analyses and experiments in cells with constitutive FZD2 expression. Furthermore, our findings indicate that Vmp1/Tmem41b depletion does not cause a global disruption of protein secretion but shows a specificity for some sub-classes of secreted proteins, such as GPCRs. GPCR secretion is tightly regulated at the ER-Golgi interface, where proper folding is essential for their route in the secretory pathway [45]. The scrambles activity of Vmp1 and Tmem41b seems crucial to support the proper maturation of FZD2, as in their absence, the receptor accumulates in ER-Golgi subdomains and is characterized by a lower stability. Structural studies have identified the binding of endogenous fatty acids as essential for the proper folding, glycosylation, and trafficking of FZD2 [50]. Based on this, we propose that depletion of Vmp1 and Tmem41b, altering the membrane composition of the ER and Golgi compartments, deprives FZD2 of lipids necessary for its maturation. This mechanism could explain how the absence of these ER-resident genes impairs activation of the WNT signaling cascade and explain the specificity for the FZD2-type receptors, as the need for fatty acid interactions has not been reported for other WNT-receptor classes [51].

Studies suggest that abnormal expression of Vmp1 and Tmem41b is associated with several pathologies, including cancer [52]. Given the involvement of WNT signaling in multiple tumor stages, clarifying how Vmp1 and Tmem41b regulate this pathway through FZD2 modulation could reveal their tumorigenic roles and identify therapeutic targets to disrupt cancer-promoting signals. The cellular systems generated in our study will help delineate the mechanisms these scramblases share in controlling protein secretion and WNT signaling. Furthermore, the role of Vmp1 and Tmem41b in modulating the WNT cascade may extend to other signaling networks, revealing other cross-connections between cellular pathways and metabolic processes.

## Methods

### Cell culture

Mouse ES cells were maintained as described in [53]. To obtain embryonic bodies (EBs), ESCs were plated on Day 1 in ESCs base media without 2i and LIF into a well of a Sphericalplate5D (Kugelmeiers). On Day 4, EBs formed and were transferred to a pHEMA-coated culture dish and cultured until Day 6. EBs were then collected and subjected to RNA extraction and RT-qPCR analysis or prepared for sectioning and immunofluorescence. For the derivation of spinal cord organoids, we followed the procedure described in [54]. XEN cells were obtained following a protocol by Niakan et al. with minor changes [36].

### CRISPR-Cas9 editing to generate mutant ESC lines

Vmp1: the gRNA (gagacgcatagcaatgagta) was cloned into a px330 vector (Addgene, #158973) and transfected in ESCs. Cells were plated by limiting dilution to derive single clones. Analysis by WB was used to identify VMP1^KO^ lines. Tmem41b: the gRNAs (gtatgtttgacctgggcgaa and gagtgacatgtggaaatcag) were cloned into px330 vectors, and KO clones were derived as described above. PCR analysis of the edited locus identified KO clones. qPCR of mRNA from Exon 2 (edited locus, N-terminal) and Exon 7 (C-terminal) further confirmed the absence of mRNA.

### Transfection and stable cell line generation

For derivation of Fzd2-HA ESCs, the PB-HA-Fzd2-IRES-Neo plasmid [53] was used. Stable expression of the construct was achieved in WT, Vmp1^KO^, and Double KO ESCs by co-transfection of the Piggybac and the PBase plasmids. Cells were plated by limiting dilution and integration events selected with G418. Multiple clones characterized by different HA-FZD2 expressions were expanded.

### Proliferation assay

Cells were collected and counted 48 h after plating. Proliferation rates were calculated as the relative factor of cells collected over cells seeded.

### LC3 staining and size characterization

Cells were plated on coverslips pre-treated with Matrigel (Corning, # 354277) in N2B27 media. The next day, samples were fixed in 4% PFA for 15 min, washed in PBS, and then treated with ice-cold MeOH for 5 min on RT. The staining was then performed as described below. Quantification was performed with the Fiji plugin TrackMate [55, 56].

### LD staining and RF microscopy

Cells were treated with or without oleic acid (200 µM - Sigma, O1008) overnight. The next day, samples were incubated with 5 µM BODIPY 493/503 (Invitrogen, #D3922) in PBS and processed for staining. Quantification was performed using the Fiji plugin TrackMate [55, 56]. For RF microscopy, cells were plated on glass bottom plates (Cellvis, # P06-1.5H-N) in N2B27 media. After 36 h, samples were treated with oleic acid o/n and then with BODIPY for 3 h. Samples were maintained in N2B27 for RF microscopy. The imaging was performed on Tomocube HT-X1 instrument at 37 C and 5% CO_2_.

### Quantification and statistical analysis

Statistical analysis was performed using Python and GraphPad Prism. Data are presented as mean-centered and the standard deviation. All experiments were repeated with at least 3 independent biological replicates unless stated otherwise. The statistical test used to evaluate significance is the Welch’s *t*-test. Statistical significance in the figures is denoted as follows: ns: *p* > 0.05, *p < 0.05, **p < 0.01, ***p < 0.001.

### Additional methods

RNA seq and Surface Proteome Profiling analysis, protocols for RNA and protein studies, and more detailed procedures can be found in Supplementary Information.

## Supplementary information

Supplemental Information

Table_S1-Gene_classes

Table_S2-ESC_DSEq2-analysis

Table_S3-XEN_DSEq2-analysis_DoubleKOvsWT

Table_S4-MS_plasma_membrane

Video S1 - Cardiomyocyte specification in WT EBs

Video S2 - Cardiomyocyte specification in DoubleKO EBs

## Data Availability

The transcriptomic data have been deposited on NCBI SRA database under the accession: PRJNA1031567. The proteomic dataset, including raw data, generated tables, and scripts used for the data analysis, is available in the PRIDE repository: PXD053039.
