# Supplementary material for: The scramblases VMP1 and TMEM41B are required for primitive endoderm specification by targeting WNT signaling
Source: Cell Death Differ. 2024 Dec 18;32(6):1086–98. doi: 10.1038/s41418-024-01435-x (PMC12162860; doi:10.1038/s41418-024-01435-x)

Supplemental Information

The scramblases VMP1 and TMEM41b are required for primitive endoderm specification by targeting WNT signaling

Markus Holzner, Tea Sonicki, Hugo Hunn, Federico Uliana, Vamshidhar R. Gade, Karsten Weis, Anton Wutz, and Giulio Di Minin

SUPPLEMENTARY METHODS

Cell Culture

Mouse ES cells were maintained as previously described in a chemically defined 2i medium with added leukemia inhibitory factor (LIF) as described in Di Minin et al. [1]. Base media composition: DMEM (Gibco, #41965039), 15% FBS (Biowest, #S1810-500) 1% sodium pyruvate (Gibco, 11360-070), 1% non-essential amino acids (Gibco, #11140-035). Lif (2000 U/mL, homemade), PD0325901 (1 µM, AxonMedchem, #391210-10-9), and Chiron (3 µM, AxonMedchem, #252917-06-9) are added fresh before use.

EB differentiation

To obtain embryonic bodies (EBs), 200k ESCs were plated on Day 1 in ESCs base media without 2i and LIF (see cell culture) into a well of a Sphericalplate5D (Kugelmeiers). On Day 4, EBs formed and were transferred to a pHEMA-coated 6cm culture dish and cultured until Day 6. EBs are then collected and subjected to RNA extraction and RT-qPCR analysis or prepared for sectioning and immunofluorescence. In brief, EBs are fixed in 4% PFA for 30 min, cryoprotected in 10%, 20%, and 30% sucrose, and then embedded and frozen in Tissue Freezing Medium (Leica, #14020108926). 15 µm sections were prepared on a cryotome, mounted onto Superfrost Plus Adhesion slides (VWR international GmBH, #631-0108) and processed for immunofluorescence staining.

Cardiomyocyte differentiation

EBs were formed as previously described. On Day 4, EBs were collected and seeded onto a non-treated 10cm dish, maintaining ESC base media. Cardiomyocytes were randomly specified and were observed as early as Day 6 of differentiation.

Spinal Cord Organoid (SCO) formation

For derivation of SCOs, ESCs were plated in EBs media (DMEM- F12 (Gibco) and Neurobasal Medium (Gibco) (1:1 ratio) supplemented with 200 mM L-glutamine and 10% of Knockout serum replacement) on Sphericalplate 5D dishes (Kugelmeiers AG) [2]. On day 2, SCOs were transferred in 10 cm tissue culture dishes and treated with RA (10 nM). SCOs were collected and processed for analysis at day 5.

Xen Differentiation

XEN cells were obtained following a protocol by Niakan et al. with minor changes [3]. In brief, 230k cells were plated in XEN medium on a gelatin-coated 3cm dish. The next day, the media was replaced with Xen medium containing 0.1 µM retinoic acid (Thermo Fischer, #17110052) and 10 ng/mL Activin A (Peprotech, #120-14). On Day 4, the medium was again replaced by XEN medium, no further supplements were added, and on Day 6, samples were either analyzed by IF or qPCR.

Rescue of double KO Xen differentiation was performed by adding 1 µM Chiron (AxonMedchem, #252917-06-9) at Day2, together with the retinoic acid and Activin A. Media was changed on day 4 to Xen medium. XEN medium composition: RPMI 1640 (Gibco, #61870010), GlutaMAX™ (Gibco, #61870036), 15% FBS (Biowest, #S1810-500), 0,1 mM ß-mercaptoethanol (Sigma, #BCCB9882).

CRISPR-Cas9 editing to generate mutant ESC lines

Vmp1: the gRNA (gagacgcatagcaatgagta) was cloned into a px330 vector (Addgene, #158973). Per 3cm well, 300k ESCs were plated and transfected with 2.5 µg of gRNA containing plasmid, as well as 0.25 µg of a GFP expressing plasmid (pCMV-GFP). GFP-positive cells were enriched by FACS and plated as single cells. Colonies were picked. Analysis by WB was used to identify identified VMP1 KO lines. Tmem41b: the gRNAs (gtatgtttgacctgggcgaa and gagtgacatgtggaaatcag) were cloned into px330 vectors, and KO clones were derived as described above. PCR analysis of the edited locus (FW: atcttggacagggggagttc RV: gaccaggggttcattgtcatt and FW: cagcacacacctttaatccagc RV: gccacatagcaagcttaagagc) identified KO clones. qPCR of mRNA from Exon 2 (edited locus, N-terminal end) and Exon 7 (C-terminal end) further confirmed the absence of mRNA.

Transfection and stable cell line generation

For derivation of *Fzd2*-HA ESCs, the PB-HA-Fzd2-IRES-Neo plasmid [1] was used. Stable expression of the construct was achieved in WT, Vmp1^{KO}, and DoubleKO ESCs by co-transfection of the Piggybac and the PBase plasmids. Cells were plated by limiting dilution and integration events selected with G418. Multiple clones characterized by different HA-FZD2 expressions were expanded. For the

downregulation of Ddit3 the following siRNA were used: siDidt3_1 rArArUrCrArArArArArCrCrUrUrCrArCrUrArCrUrCrUrUGA, siDidt3_2 rGrArCrGrGrUrArCrArUrUrGrUrUrArUrUrArCrUrGrUCC, and siDidt3_3 rArArCrArGrArGrGrUrCrArCrArCrGrCrArCrArUrCrCrCAA. Negative Control DsiRNA (IDT, 51011404) was used as a control. Cells were transfected with Lipofectamine™ RNAiMAX Transfection Reagent.

Proliferation assay

100k ESCs were plated in the well of an MW12. 48 hours later, cells were collected and counted. Proliferation rates were calculated as the relative factor of cells collected over cells seeded.

LC3 staining and size characterization

100k cells were plated on cover-slips, pre-treated with Matrigel (Corning, # 354277), in N2B27 media. N2B27 composition: 50 mL Neurobasal media (Gibco, # 2348949), 50 mL Advanced DMEM F12 (Gibco, # 12634010), 4% BSA (Gibco, # 15260), 1% L-Glutamine (Gibco, # 25030024), 1% B27 supplement (Gibco, # 2336837), 0,5% N2 supplement (Gibco, # 2328253), and 0,1 mM ß-mercapotethanol (Sigma, #BCCB9882). The next day, samples were fixed in 4% PFA for 15 min, washed 3 times with PBS, and then treated with ice-cold MeOH for 5 min on RT. The staining was then performed as described below. Quantification was performed with the Fiji plugin TrackMate [4, 5] to automatically detect puncta with a diameter of 1, 2, and 3 microns at a threshold of 600, 200, and 150, respectively. Nuclei were detected at an average size of 15 microns with a threshold of 10. Unique particles were annotated for 5 different frames for each condition and shown as particles per cell. The experiment was repeated 3 times.

LD staining and RF microscopy

Samples were plated as for the LC3 staining. For samples treated with oleic acid, 200 µM (Sigma-Aldrich, # O1008-1G) were added at plating overnight. The next day, samples were incubated in 5 µM BODIPY 493/503 (Invitrogen, #D3922) in PBS and subsequently washed 3 times with PBS and fixed in 4 % PFA for 15 min. Samples were then washed, stained with Dapi for 15 min at RT, and mounted with Mowiol.

Quantification was performed using the Fiji plugin TrackMate [4, 5] detecting droplets of 1, 2, and 3 microns in diameter at a threshold of 900. Nuclei were detected at 10 microns and a threshold of 1.

For RF microscopy, 250k cells were plated per well of a 6-well glass bottom plate (Cellvis, # P06-1.5H-N) in N2B27 media. After 36 h, samples were treated with 200 µM oleic acid o/n and then with BODIPY for 3 h. Samples were washed 3 times with PBS and maintained in N2B27 for RF microscopy. The imaging was performed on Tomocube HT-X1 instrument at 37C and 5 % CO2. At least 5-8 images were acquired for each condition for each well. The z-stacks were acquired with 1.06 µm slices. Max Intensity projections (MIP) are generated on the Tomostudio 3.3.9. The fluorescence max intensity projections were resized to Refractive index tomograms for comparison. The ROIs from each image were isolated for further analysis. Comdet plugin was used for identifying high refractive index regions. The approximate particle size was 2 pixels and Intensity threshold values were chosen individually for each image to avoid any false positives or negatives.

Immunofluorescence

Samples were washed 3 times with PBS, fixed in 4% PFA for 10 min and again washed 3 times with PBS. Samples stained for LC3 were then treated for 5 min with 100% MeOH at RT and washed again 3 times with PBS. Samples were then blocked and permeabilized with 10% donkey serum (Jackson Immunoresearch, #017-000-121) and 0,1% Triton X (Thermo Fischer, # HFH10) in PBS for 20 min and incubated with primary antibodies at 4 °C over-night. Oct4 (1:100, Satna Cruz, # sc-5279), Nanog (1:100, Abcam, # ab80892), Sox2 (1:50, R&D Systems #AF2018), Lc3 (1:200, Cell Signalling, # 2775S), Pdi (1:200, Cell Signalling, # 3501S), Rcas1 (1:200, Cell Signalling, # 12290S), Rab7 (1:200, Cell Signaling, # 9367S) Sox1 (1:50, R&D Systems, # AF3369), Sox17 (1:200 R&D Systems, # AF1924-SP), Gata4 (1:100 R&D Systems, # AF1700-SP), Gata6 (1:100 R&D Systems, # AF1700-SP), Dab2 (1:100 BD, # 610464). The next day, samples were washed 3 times with 0.1% Triton X in PBS and then incubated with secondary antibodies and Dapi.

Western Blot Analysis

Cells were lysed in membrane buffer (150 mM NaCl, 50 mM Tris-HCl pH 7,5, 1 mM 2-mercaptoethanol (Sigma, #M3148) and 1% CHAPS (Sigma, #C3023) in water).

Lysates were run on SDS-Page and transferred to nitrocellulose membranes. Bands were detected with the Amersham ECL Prime Western Blotting Detection Reagents (#RPN2232) and captured on Fujifilm Super RX-N films (#47410). Antibodies for Vmp1 (Cell Signalling, #12929S) ,DDIT3 (Cell Signalling, #2895T), β-CATENIN (Santa Cruz, sc-7963), β-ACTIN (BioLegend, W16197A), E-CADHERIN (BioLegend, 67A4), H3 (Abcam, #1791), HA-epitope (Roche, 11867423001), HSP90 (Santa Cruz, #sc-101494) were used. The Quantification of western blot bands was performed with ImageJ.

RNA extraction and RT qPCR

RNA was extracted with the RNeasy Kit (Quiagen, #74104) following the manufacturer's instructions, including the on-column DNA digestion. EB and SCO samples were pre-treated with the QIAshredder (Quiagen, #79656). cDNAs were prepared using the Bio-Rad iScript Reverse Transcription Supermix for RT-qPCR (#1708841). RT qPCR samples were prepared using the KAPPA SYBR R fast (Roche, KK4611). Primer Sequences:
Tmem41b Exon2 FW: TGCAGAAGCTGGATCAGCAA, RV: GAGCCTTGGCGTCATCCATA
Tmem41b Exon7 FW: GCAGGTAGAGCGTCACAGAG, RV: TGCAGAGTTGTTCCTGCCTT
Sdha FW: CGTTATGTGAGGGGTGTGCCTT, RV: CCTTACCAAACCTTGTGTCTGGA
Sox1 FW: ACGTAGCCAACGTAGACAC, RV: ATGAGCGTCGTCCCGTGG
Fgf5 FW: GCCTGTCCTTGCTCTTCCTCAT, RV: GGAGAAGCTGCGACTGGTGA
Actc1 FW: TGTAGGACCGTGTCGAAACC, RV: CGAAACCACACACTGTTACCG
Brachyury FW: TCTCTGGTCTGTGAGCAATGGT, RV: TGCGTCAGTGGTGTGTAATGTG
Gata6 FW: GAAGCGCGTGCCTTCATC, RV: GTAGTGGTTGTGGTGTGACAGTTG
Dab2 FW: TTGATGATGTGCCTGATGCT, RV: TTTGCTTGTGTTGTCCCTGA
Sox17 FW: CCCAACACTCCTCCCAAAGTATC, RV: TTCCCTGTCTTGGTTGATTTCT
Ddit3 FW: GGCTCAAGCAGGAAATCGAG, RV: AAGTGAGAGGCTGTTGACAC

Quantification and statistical analysis

Statistical analysis was performed using Python and GraphPad Prism. Data are presented as mean-centered and the standard deviation. All experiments were repeated with at least 3 independent biological replicates unless stated otherwise. The statistical test used to evaluate significance is the Welch's *t* test. Statistical significance in the figures is denoted as follows: ns: $p > 0.05$, *: $p < 0.05$, **: $p < 0.01$, ***: $p < 0.001$.

RNA seq

RNA samples were submitted to the Functional Genomics Center in Zurich (FGCZ) for RNA sequencing. The quality of the RNA was determined with a Fragment Analyzer standard sensitivity RNA measurement (SS RNA kit (15 nt), Agilent, Waldbronn, Germany). The measured concentrations (> 50 ng/µl) and RIN (>9.9) values qualified for a Poly-A enrichment strategy in order to generate the sequencing libraries applying the TruSeq mRNA Stranded Library Prep Kit (Illumina, Inc, California, USA). After Poly-A selection using Oligo-dT beads the mRNA was reverse-transcribed into cDNA. The cDNA was fragmented, end-repaired and polyadenylated before ligation of TruSeq UD Indices (IDT, Coralville, Iowa, USA). The quality and quantity of the amplified sequencing libraries were validated using a Fragment Analyzer SS NGS Fragment Kit (1–6000 bp) (Agilent, Waldbronn, Germany). The equimolar pool of samples was spiked into a NovaSeq6000 run targeting 20M reads per sample on a S1 FlowCell (Novaseq S1 Reagent Kit, 100 cycles, Illumina, Inc, California, USA). Reads from RNA-seq were first preprocessed by Trimmomatic (v0.40) [6] to remove low-quality bases and adapters. Then, reads were aligned to mouse genome GRCm38 (release 81 version from Ensembl) using HISAT2 (v2.2.1) [7]. HTSeq Count (v2.0.2) [8] was used to count reads for each gene (Ensembl GRCm38.81), ignoring reads on overlapped regions. Differentially expressed genes were defined by DESeq2 (v1.40.2) [9]. MA plot was generated using custom-made scripts. Genes significantly enriched show a fold change > 1 and FDR (adjusted p-value) < 0.01. The read counts were transformed in RPKM for the heatmap representations of specific genes and pathways. The genes corresponding to germ-layer differentiation markers (including those for embryonic and extra-embryonic endoderm), biological processes, and signaling pathways were obtained through literature mining and the KEGG database (Table S1). The processed data are available as excel files (Table S2 and Table S3)

and raw data have been deposited on NCBI SRA database under the accession: PRJNA1031567

Surface Proteome Profiling

Sample preparation and biotin labeling were performed following the instructions from [10, 11]. In brief, 10M cells were collected per sample using Accutase (Biolegend, #423201). Cell pellets were washed once with PBS and then incubated, resuspended in 1 mL biotinylation buffer (97 µM aminooxy-biotin, Biotium #90113, 1 mM sodium periodate, Thermo-scientific #20504, 0,093% aniline, Sigma-Aldrich, #242284) for 30 min at 4 °C. Next, cells were pelleted, the reaction quenched with 1 mM glycerol (30min, 4 °C) and washed once with PBS and lysed in 1 mL of lysis buffer (see western blot) by sonication (Dr. Hielscher sonicator, 30 sec at 80% amplitude and 80% cycle time). Lysates were pelleted, the supernatant transferred into a fresh tube, and incubated with 30 µL (dry resin) of streptavidin agarose resin (Thermo-Scientific, #20347) for 1.5h at 4 °C. beads collected, transferred onto a column (BioRad, #7326008) and washed with 8 mL of lysis buffer and 4 mL of 0,5% SDS in 50 mM TEAB. Beads were resuspended in 200 µL 50 mM Ambic with 10 mM TCEP (30 min, 37 °C), then 20 mM iodoacetamide was added (30 min. 37 °C). Beads were further washed with 4 mL of SDS buffer (see above), 8 mL Urea buffer (6M Urea in 50 mM TEAB, pH=8,5), and 2 mL of 50 mM TEAB. Beads were taken up in 200 µL TEAB buffer and digested with 3 µL Trypsin (Promega) overnight, 37 C. Tryptic digestion was quenched by the addition of acetonitrile (ACN). Tandem mass tag (TMT) isobaric reagents (TMTpro 6plex Thermo Fisher Scientific) were dissolved in anhydrous ACN to a final concentration of 20 mg/ml, of which a unique TMT label was added (100µg). Peptides were incubated at room temperature for one hour, TMT labeling reactions were halted by 0.3% hydroxylamine and dried. Peptides were fractionated by high pH fraction in reverse phase (microspin column, Nest group) following the procedure based on the high pH reversed-phase peptide fractionation kit (Pierce).

LC-MS/MS was performed on an Orbitrap Exploris 480 mass spectrometer (Thermo Fisher) coupled to a Vanquish Neo liquid chromatography system (Thermo Fisher). Peptides were separated using a reverse phase column (75 µm ID x 400 mm New Objective, in-house packed with ReproSil Gold 120 C18, 1.9 µm, Dr. Maisch GmbH) across 180 min linear gradient from 7 to 35% (buffer A: 0.1% [v/v] formic acid; buffer

B: 0.1% [v/v] formic acid, 80% [v/v] acetonitrile). Samples were acquired in DDA mode (Data Dependent Acquision) with MS1 scan (scan range = 350-1500, R=120K, max injection time auto and AGC target = 100), followed by dependent MS2 scans (first mass (m/z) 110, R = 45K, max injection time auto and AGC target = 200).Cycle time was set to 2 sec. Peptides with a charge between 2-5 were isolated (m/z = 0.7) and fragmented (NCE 36%).

Acquired spectra were searched using the MaxQuant software version 2.1.0.0 against *mus musculus* proteome reference dataset (http://www.uniprot.org/, downloaded on 03.04.2020) extended with reverse decoy sequences. The search parameters were set to tryptic peptides, maximum two missed cleavage, carbamidomethyl as static peptide modification, oxidation (M) and deamidation (N-terminal), and TMT6 as static peptide modification. The MS and MS/MS mass tolerance was set to 10 ppm. A false discovery rate of < 1% was used at PSM and protein levels. The abundance of 1417 proteins was determined from the intensity of the top two peptides. Intensity values were normalized by the median, and missing values were imputed using random sampling from a normal distribution generated from 1% less intense values. Statistical analysis was performed using unpaired two-sided t-test, and p-values were corrected using Benjamin-Hochberg correction. The entire dataset, including raw data, generated tables, and scripts used for the data analysis, is available in the PRIDE repository: PXD053039. Matrices with protein intensities are reported in Table S4.

SUPPLEMENTARY REFERENCES

1. Di Minin, G., et al., *TMED2 binding restricts SMO to the ER and Golgi compartments.* PLoS Biol, 2022. **20**(3): p. e3001596.
2. Holzner, M., A. Wutz, and G. Di Minin, *Applying Spinal Cord Organoids as a quantitative approach to study the mammalian Hedgehog pathway.* PLoS One, 2024. **19**(6): p. e0301670.
3. Niakan, K.K., et al., *Derivation of extraembryonic endoderm stem (XEN) cells from mouse embryos and embryonic stem cells.* Nat Protoc, 2013. **8**(6): p. 1028-41.
4. Ershov, D., et al., *TrackMate 7: integrating state-of-the-art segmentation algorithms into tracking pipelines.* Nat Methods, 2022. **19**(7): p. 829-832.
5. Tinevez, J.Y., et al., *TrackMate: An open and extensible platform for single-particle tracking.* Methods, 2017. **115**: p. 80-90.
6. Bolger, A.M., M. Lohse, and B. Usadel, *Trimmomatic: a flexible trimmer for Illumina sequence data.* Bioinformatics, 2014. **30**(15): p. 2114-20.
7. Kim, D., et al., *Graph-based genome alignment and genotyping with HISAT2 and HISAT-genotype.* Nat Biotechnol, 2019. **37**(8): p. 907-915.

8. Putri, G.H., et al., *Analysing high-throughput sequencing data in Python with HTSeq 2.0.* Bioinformatics, 2022. **38**(10): p. 2943-2945.
9. Love, M.I., W. Huber, and S. Anders, *Moderated estimation of fold change and dispersion for RNA-seq data with DESeq2.* Genome Biol, 2014. **15**(12): p. 550.
10. Wojdyla, K., et al., *Cell-Surface Proteomics Identifies Differences in Signaling and Adhesion Protein Expression between Naive and Primed Human Pluripotent Stem Cells.* Stem Cell Reports, 2020. **14**(5): p. 972-988.
11. Weekes, M.P., et al., *Proteomic plasma membrane profiling reveals an essential role for gp96 in the cell surface expression of LDLR family members, including the LDL receptor and LRP6.* J Proteome Res, 2012. **11**(3): p. 1475-84.

SUPLEMENTAL TABLES

The following Excel files have been included as supplementary material:

Table S1. Gene classes

Genes categorized by GO terms for RNAseq data analysis as displayed in Fig. 1, 3, S3, and S5.

Table S2. ESC DSEq2 analysis

Results of DSEq2 analysis performed on the transcriptomic profiles of ESCs WT, Vmp1, Tmem41b, and DoubleKO. Folds are shown relatively to WT cells.

Table S3. XEN DSEq2 analysis DoubleKOvsWT

Results of DSEq2 analysis performed on the transcriptomic profiles of XEN WT and DoubleKO. Folds are shown relatively to WT cells.

Table S4. MS plasma membrane

Results of MS analysis of plasma membrane proteins detected in WT and DoubleKO cells

SUPLEMENTAL FIGURE LEGENDS

S1 Characterization of single and double mutant ESCs

(A) Efficient depletion of VMP1 protein in Vmp1 and Double KO ESCs. Western Blot analysis shows VMP1 expression and HSP90 as housekeeping. **(B)** PCR analysis of the deletion induced by CRISPR-Cas9 at the Tmem41b genomic locus in WT and mutant cells. WT cells show amplification of a product of 3000 bps; in Tmem41b and Double KO ESCs, a fragment of 500 bps is detected. The PCR negative control (C-) is indicated. For each mutation, two independent clones were characterized. **(C-D)** RT-qPCR analysis showing decreased Tmem41b expression in Tmem41b and Double KO ESC using exon-spanning primers specific for exon 2 **(C)** and exon 7 **(D)**. mRNA levels are relative to WT ESC sample and normalized to Sdha mRNA. **(E)** Single and Double KO ESCs show similar proliferation rate. Plotted are proliferation factors after

48h. Each dot represents independent experiments. For each KO condition, two independent clones were tested. **(F-G)** Single and double KO ESCS show similar expression of pluripotency markers. **(F)** Representative IF images showing WT and mutant ESCs stained for the pluripotency markers NANOG, OCT4, and SOX2. Nuclei are stained with Dapi. Scale bar: 20 µm. **(G)** Quantification of pluripotency markers from **F** relative to the total cell number. Each dot represents an independent frame analyzed. **(H)** Morphology of single and double KO ESCs is like WT ESCs. Brightfield images showing ESCs morphology. Scale bar: 100 µm. **(I-J)** Transcriptomic analysis of VMP1 **(I)** and TMEM41b **(J)** KO ESCs showed only limited transcriptional differences, plotted here as MA plot. Genes differentially expressed are labeled in blue. Genes highlighted in red are pluripotency markers.

S2 Characterization of Autophagy and Lipid Droplet phenotypes

(A) Single and Double KO ESCs show accumulation of LC3+ autophagosomes. Representative IF images show ESCs stained for LC3; nuclei are stained with Dapi. Scale bar: 20 µm. **(B)** Single and Double KO ESCs show increased number of autophagosomes per cell. Graph shows quantification of LC3 puncta per cell of (A) in WT, Vmp1, Tmem41b, and double KO ESCs. Each dot represents an analyzed frame. Two independent mutants were analyzed per KO condition. **(C)** Autophagosomes in double KO ESCs are bigger than in single KO conditions. Pie charts show the size distribution of LC3 puncta of 1, 2, and 3 µm diameter in each mutant condition. Two independent mutants were analyzed per mutant condition. **(D)** Double KO ESCs accumulate LD. Live cell staining of WT, Vmp1, Tmem41b, and double KO ESCs with BODIPY. Cells were pre-treated with or without oleic acid (OA). **(E)** Quantification of LD frequency per cell in the absence or in the presence of OA treatment. **(F)** LDs are visible by Refractive index microscopy. Representative image of Double KO ESCs treated with OA highlighting a calibration bar for refractive index. White spots correspond to a refractive index of 1.4, which is specific for LDs. Scale bar: 20 µm. **(G)** LDs in refractive index microscopy overlay with BODIPY staining. Representative images show RF images co-stained with BODIPY (bottom panel). Scale bar: 10 µm.

S3 Vmp1, Tmem41b, and Double KO ESCs show similar morphology and transcriptional profile

(A) Single and double KO ESCs show similar morphology of intracellular organelles. Representative confocal IF shows ESCs stained for RCAS1 (Golgi, left), RAB7 (late endosomes, center), and PDI (ER, right) in green; nuclei were stained with Dapi. Scale bar: 20 µm. **(B-E)** Transcriptional analyses of WT and Vmp1, Tmem41b, and Double KO ESC show no significantly deregulated genes. **(B)** PCA analysis of the transcriptional profile of WT, single, and Double KO ESCs. **(C)** Diagram showing uniquely and collectively differentially expressed genes in Vmp1, Tmem41b, and Double KO ESCs. **(D)** Differentially expressed genes in single and Double KO ESCs are not enriched for specific developmental processes. The plot illustrates the differentially expressed genes based on germ layers, with the size of dots indicating the amount of differentially expressed genes. **(E)** Differentially expressed genes in single and double KO ESCs are not enriched for specific signaling pathways. The plot illustrates the differentially expressed genes based on molecular pathways, with the size of dots indicating the amount of differentially expressed genes.

S4 Vmp1, Tmem41b and Double KO ESCs differentiate into the three germ layers, but not into the Dab2 positive primitive endoderm

(A) ESCs differentiated to EBs express lineage markers for ectoderm (Fgf5), mesoderm (Brachyury). They show decreased expression of the pluripotent marker Pouf51. Graphs show mRNA levels of WT ESCs and EBs normalized to the Sdha gene. Each dot represents an independent experiment. **(B)** RT-qPCR analysis for Fgf5 and Brachyury mRNA expression in mutant EBs relative to WT EBs. Two independent clones were analyzed per cell line. Each dot represents an independent experiment. The line at 0,7 indicates an arbitrary threshold after which successful expression of the marker is considered. **(C)** The heatmap summarizes the expression of a given marker across all experiments and compares it to the WT EBs expression profile by calculating p values. (**D)** Double KO ESCs differentiate into SOX1 expressing Spinal Cord organoids (SCO). Representative IF images of SCOs stained for the neuronal stemness marker SOX1; nuclei are stained with Dapi. Scale bar: 50 µm.

S5 Double KO ESCs show a delay in XEN cell specification

(A-B) Double KO ESCs differentiate at low efficiency into XEN cells. **(A)** Graphs show mRNA levels of WT and Double KO XEN at day 6. Data were normalized to the Sdha gene and expressed relative to mRNA level detected in WT ESCs. Each dot represents an independent experiment. **(B)** Representative IF images of day 6 XEN cells stained for the XEN markers DAB2 and GATA6 in the left panel and GATA4 and SOX17 in the right panel; nuclei are stained with Dapi. Scale bar: 150 µm. **(C)** XEN cells derived from Double KO ESCs can be maintained in culture. Brightfield images show WT and Double KO XEN cells at passage 3. Scale bar: 50 µm **(D-F)** Transcriptional analysis of XEN cells. **(D)** Differentially expressed genes in Double KO vs. WT conditions do not cluster by biological processes. Plot shows differentially expressed genes over biological processes. The size of dots corresponds to the number of differentially expressed genes. **(E)** Double KO XEN cells show decreased expression of XEN markers. Heatmap comparing min to max expression of XEN markers in WT and double KO XEN cells.

S6 Analysis of protein abundance at the plasma membrane in WT and VMP1^{KO} cells.

(A) Heatmap depicting sample correlation in the MS analysis. **(B)** GO term analysis comparing protein enrichment between plasma membrane-purified samples and whole-cell lysates. **(C)** Volcano plot illustrating differentially expressed proteins at the plasma membrane in WT and VMP1KO ESCs. Gray lines indicate the selection criteria: a log2 fold-change > 1 and a false discovery rate < 0.01. Proteins meeting this criteria are highlighted in red. The positions of Frizzled proteins are annotated. **(D)** mRNA expression levels of Frizzled genes in WT and VMP1KO ESCs, as shown in the transcriptomic profile described in Fig. 1. **(E)** Western blot analysis of HA-FZD2 abundance at the PM in WT and TMEM41b^{KO} ESCs. E-CADHERIN and ACTIN serve as controls for PM purification and loading, respectively. **(F)** Quantification of HA-FZD2 protein levels at the PM across three biological replicates relative to WT cells and normalized to the total HA-FZD2 (Input). Data are represented as mean ± SD.

S7 WNT signaling upregulation rescues the defect in XEN specification of Double KO cells.

(A) Cytoplasmic β-CATENIN levels are increased by CHIR treatment. Western blot analysis was performed on whole-cell lysates (1/50) and purified cytoplasmic fractions

(1/50) from WT cells treated with CHIR during the differentiation process (2 days). Histone H3, and ACTIN were used as controls for purification and loading. **(B)** Expression of WNT target genes in WT and Double KO cells upon CHIR treatment. Graphs show relative Wnt3 and Dkk1 mRNA levels normalized to the Gapdh gene. Dots represent biological independent experiments. Data are represented as mean ± SD. **(C-D)** Chiron-dependent WNT activation rescues the delay of Double KO XEN differentiation. **(C)** Brightfield images show XEN clusters of Double KO XEN cells (highlighted by white lines) with and w/o Chiron treatment. Scale bar: 100 μm. IF staining for DAB2 and GATA6 **(D)**; nuclei are stained with Dapi. Scale Bar: 150 μm.

S8 Over-expression of FZD2 rescues the defect in XEN specification of Double KO cells.

(A) Derivation of ESC clones with HA-FZD2 over-expression. Western blot showing HA-FZD2 expression in WT and Double KO cells. ACTIN is used as a loading control. **(B)** Expression of WNT target genes in WT and Double KO cells overexpressing FZD2. Graphs show relative Wnt3 and Dkk1 mRNA levels normalized to the Gapdh gene. Dots represent biological independent experiments. Data are represented as mean ± SD. **(C-D)** FZD2 overexpression rescues the differentiation delay in Double KO XEN cells. Immunofluorescence staining for SOX17 and GATA4 or DAB2 and GATA6 **(C)** in WT and Double KO cells. Nuclei are counterstained with DAPI. Scale bar: 100 μm. **(D)** Quantification of GATA6 and DAB2 positive cells during XEN differentiation, expressed as a percentage of total cells (DAPI-stained).

S9 FZD2 maturation is impaired in Double KO cells

(A) Silencing of Atg2 mimics the effect of the depletion of Vmp1 and Tmem41b. Expression of WNT target genes and XEN markers in WT and Double KO cells upon Atg2 down-regulation. Graphs show relative Dkk1 and Gata4 mRNA levels normalized to the Gapdh gene. RNA interference efficiency is evaluated by analyzing mRNA expression levels of Atg2a and Atg2b. Dots represent biological independent experiments. Data are represented as mean ± SD. **(B)** Quantification of total FZD2 stability in WT and Double KO cells. Dots represent biological independent experiments. Data are represented as mean ± SD.

S10 Uncropped original western blots used in the manuscript

Figure S1

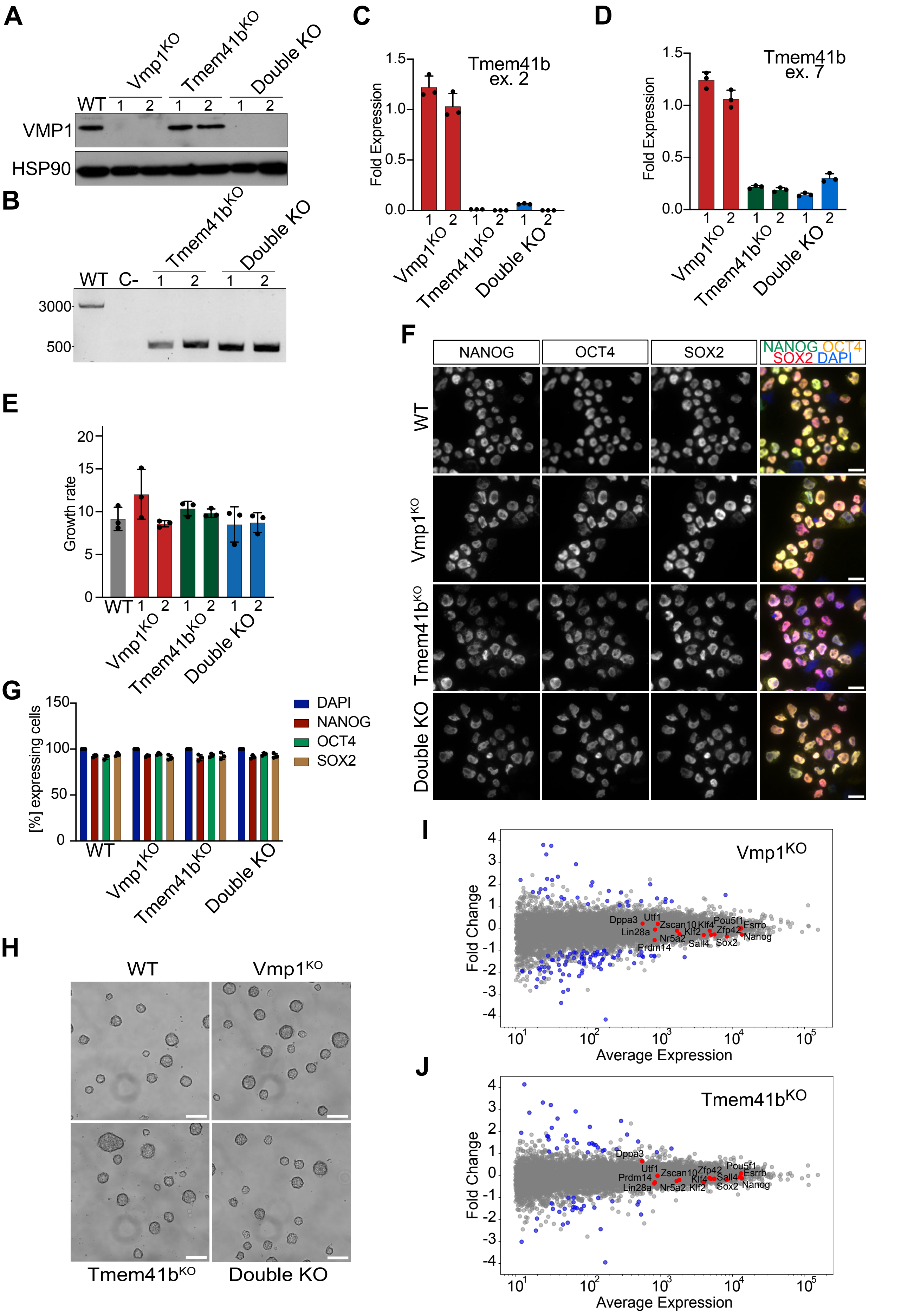
A
Vmp1KO
Tmem41bKO
Double KO
WT 1 2 1 2 1 2
VMP1
HSP90
B
Tmem41bKO
Double KO
WT C- 1 2 1 2
3000
500
C
Tmem41b ex. 2
Fold Expression
Vmp1KO
Tmem41bKO
Double KO
D
Tmem41b ex. 7
Fold Expression
E
Growth rate
WT
F
NANOG
OCT4
SOX2
NANOG OCT4 SOX2 DAPI
G
[%] expressing cells
DAPI
NANOG
OCT4
SOX2
H
WT
Vmp1KO
Tmem41bKO
Double KO
I
Vmp1KO
Fold Change
Average Expression
Dppa3
Utf1
Zscan10
Klf4
Pou5f1
Esrrb
Lin28a
Klf2
Zfp42
Nr5a2
Nanog
Prdm14
Sall4
Sox2
J
Tmem41bKO
Fold Change
Average Expression

Figure S2

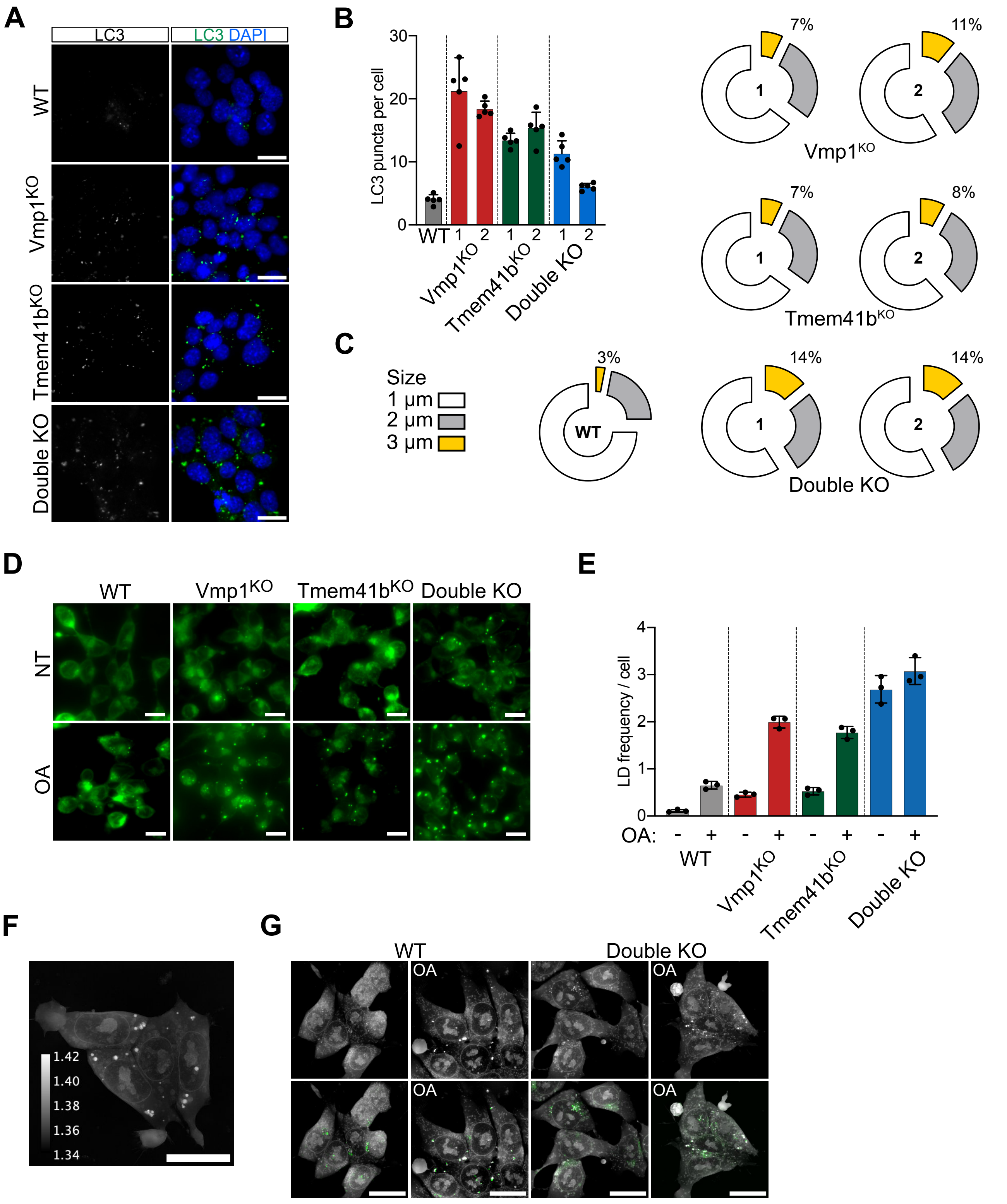
A
LC3
LC3 DAPI
WT
Vmp1KO
Tmem41bKO
Double KO
B
LC3 puncta per cell
30
20
10
0
WT
1
2
Vmp1KO
Tmem41bKO
Double KO
7%
11%
Vmp1KO
7%
8%
Tmem41bKO
C
Size
1 µm
2 µm
3 µm
3%
WT
14%
14%
Double KO
D
WT
Vmp1KO
Tmem41bKO
Double KO
NT
OA
E
LD frequency / cell
4
3
2
1
0
OA:
-
+
WT
Vmp1KO
Tmem41bKO
Double KO
F
1.42
1.40
1.38
1.36
1.34
G
WT
Double KO
OA

Figure S3

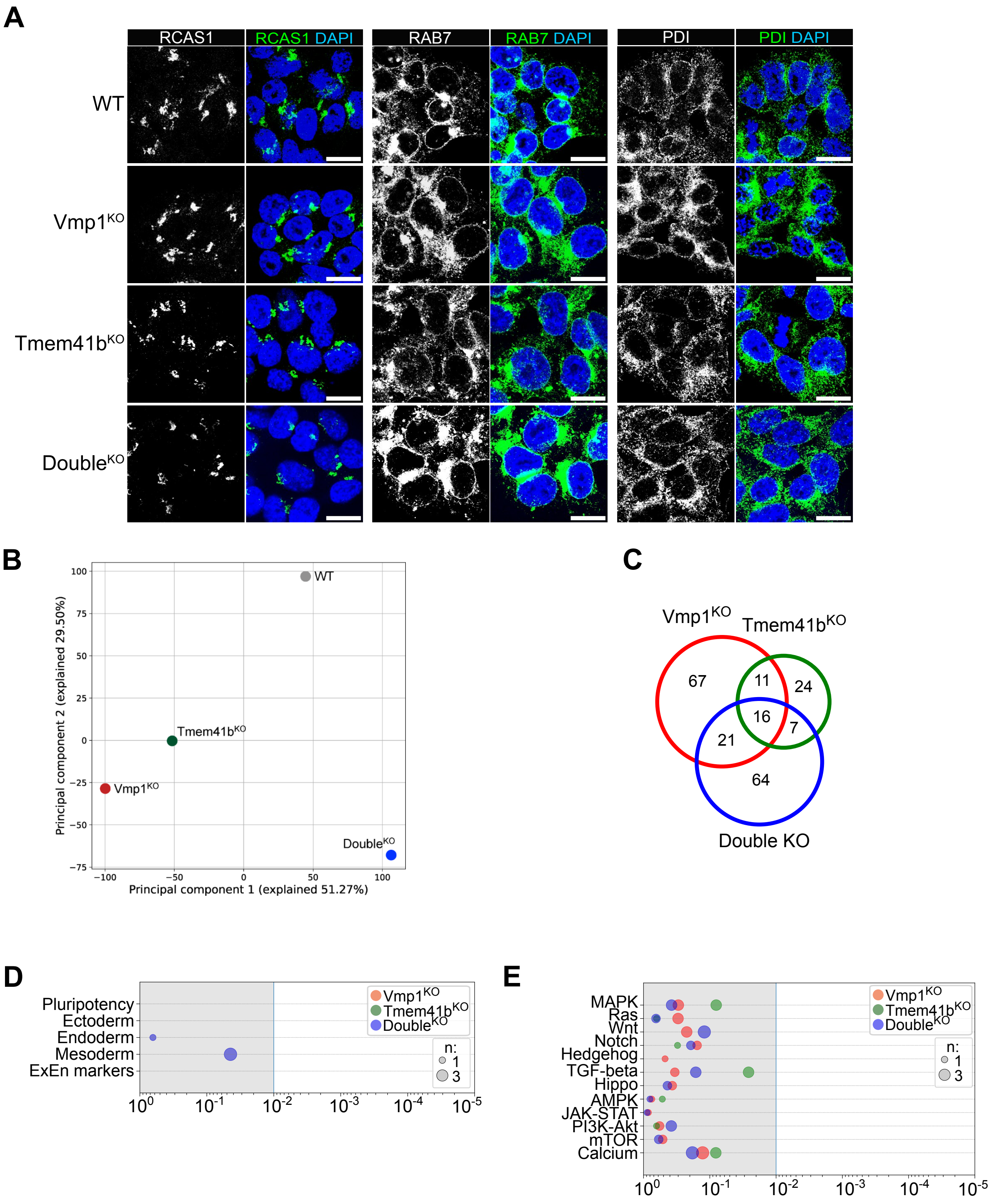
A
RCAS1
RCAS1 DAPI
RAB7
RAB7 DAPI
PDI
PDI DAPI
WT
Vmp1KO
Tmem41bKO
DoubleKO
B
Principal component 1 (explained 51.27%)
Principal component 2 (explained 29.50%)
WT
Tmem41bKO
Vmp1KO
DoubleKO
C
Vmp1KO
Tmem41bKO
Double KO
67
11
24
16
7
21
64
D
Pluripotency
Ectoderm
Endoderm
Mesoderm
ExEn markers
Vmp1KO
Tmem41bKO
DoubleKO
n:
1
3
E
MAPK
Ras
Wnt
Notch
Hedgehog
TGF-beta
Hippo
AMPK
JAK-STAT
PI3K-Akt
mTOR
Calcium

Figure S4

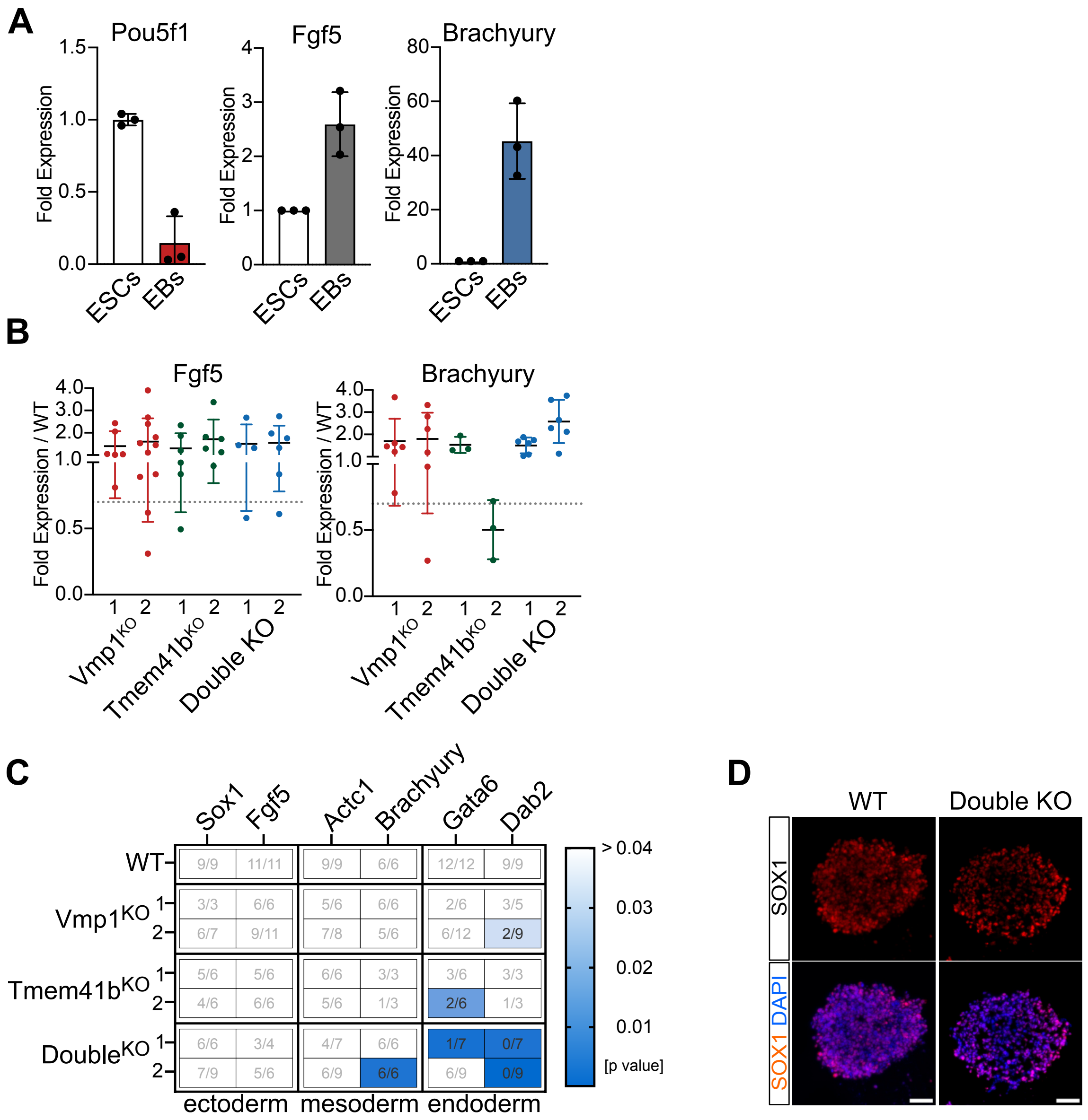
A
Pou5f1
Fgf5
Brachyury
Fold Expression
ESCs
EBs
B
Fgf5
Brachyury
Fold Expression / WT
Vmp1KO
Tmem41bKO
Double KO
C
Sox1
Fgf5
Actc1
Brachyury
Gata6
Dab2
WT
Vmp1KO
Tmem41bKO
DoubleKO
9/9 11/11 9/9 6/6 12/12 9/9
3/3 6/6 5/6 6/6 2/6 3/5
6/7 9/11 7/8 5/6 6/12 2/9
5/6 5/6 6/6 3/3 3/6 3/3
4/6 6/6 5/6 1/3 2/6 1/3
6/6 3/4 4/7 6/6 1/7 0/7
7/9 5/6 6/9 6/6 6/9 0/9
ectoderm
mesoderm
endoderm
> 0.04
0.03
0.02
0.01
[p value]
D
WT
Double KO
SOX1
SOX1 DAPI

Figure S5

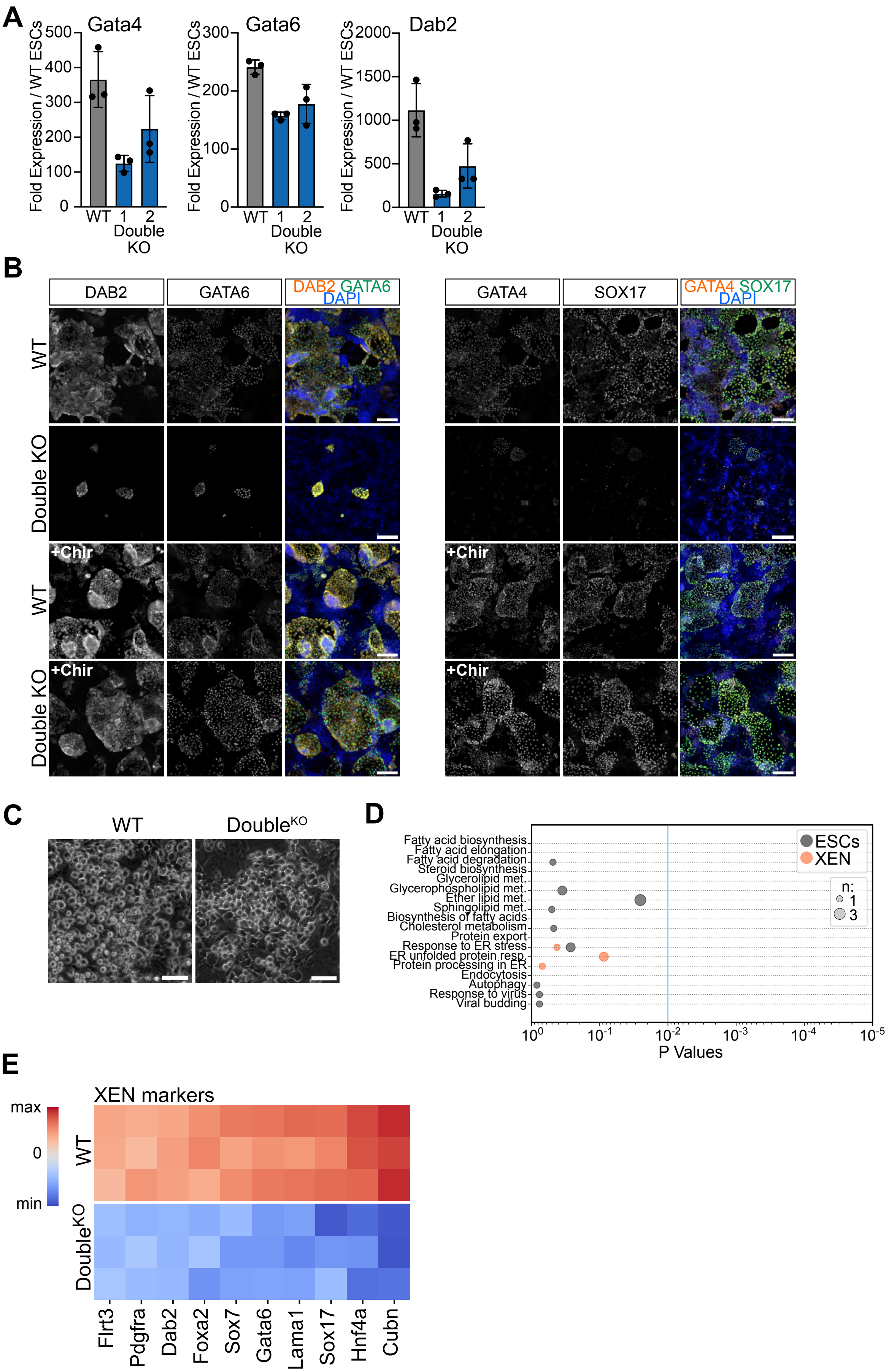

A
Gata4
Gata6
Dab2
Fold Expression / WT ESCs
WT 1 2
Double KO
B
DAB2
GATA6
DAB2 GATA6 DAPI
GATA4
SOX17
GATA4 SOX17 DAPI
WT
Double KO
+Chir
C
WT
Double^{KO}
D
Fatty acid biosynthesis
Fatty acid elongation
Fatty acid degradation
Steroid biosynthesis
Glycerolipid met.
Glycerophospholipid met.
Ether lipid met.
Sphingolipid met.
Biosynthesis of fatty acids
Cholesterol metabolism
Protein export
Response to ER stress
ER unfolded protein resp.
Protein processing in ER
Endocytosis
Autophagy
Response to virus
Viral budding
ESCs
XEN
n:
1
3
P Values
E
XEN markers
max
0
min
WT
Double^{KO}
Flrt3
Pdgfra
Dab2
Foxa2
Sox7
Gata6
Lama1
Sox17
Hnf4a
Cubn

Figure S6

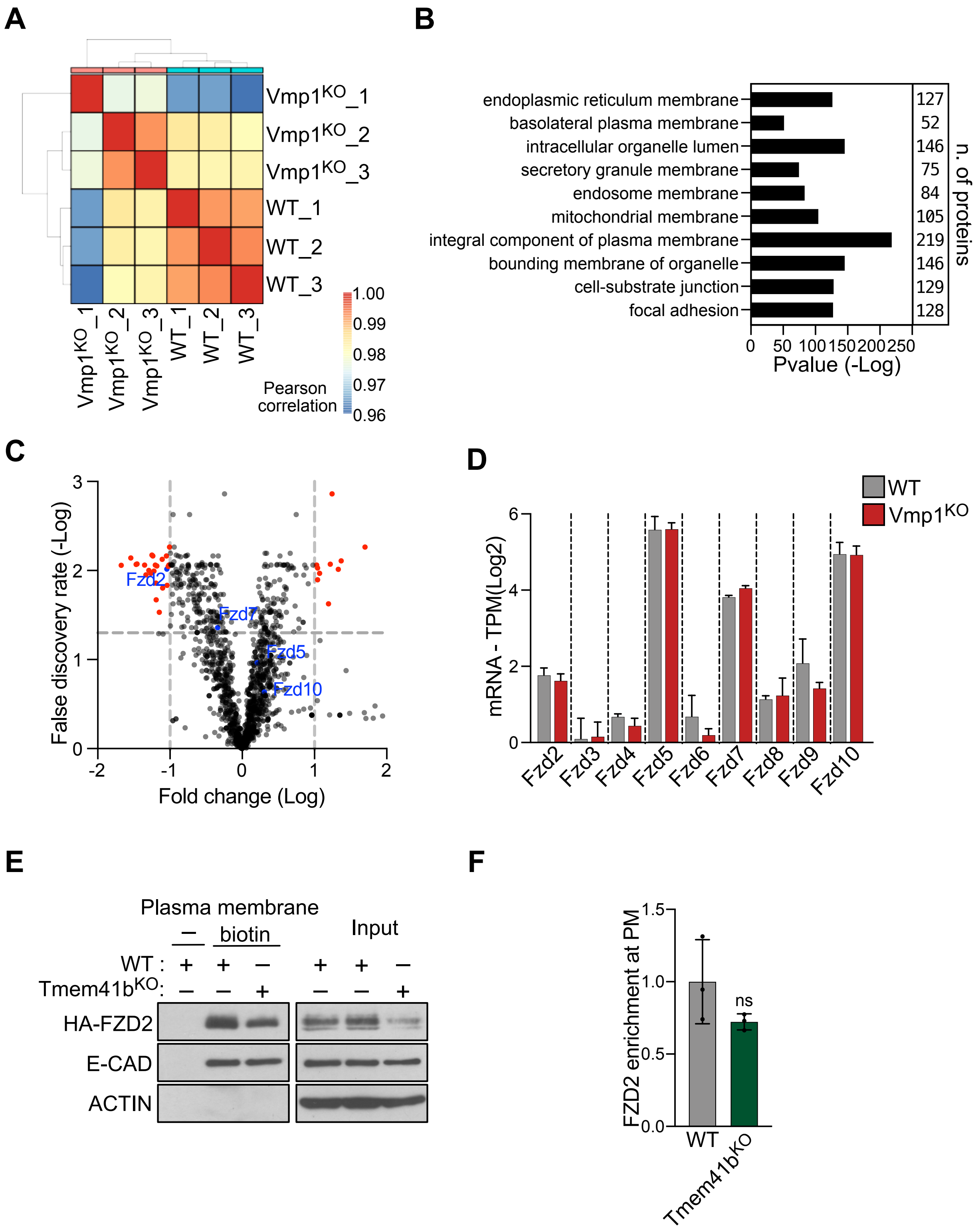
A
B
C
D
E
F
Vmp1KO_1
Vmp1KO_2
Vmp1KO_3
WT_1
WT_2
WT_3
Pearson correlation
1.00
0.99
0.98
0.97
0.96
endoplasmic reticulum membrane
basolateral plasma membrane
intracellular organelle lumen
secretory granule membrane
endosome membrane
mitochondrial membrane
integral component of plasma membrane
bounding membrane of organelle
cell-substrate junction
focal adhesion
127
52
146
75
84
105
219
146
129
128
n. of proteins
Pvalue (-Log)
False discovery rate (-Log)
Fold change (Log)
Fzd2
Fzd7
Fzd5
Fzd10
WT
Vmp1KO
mRNA - TPM(Log2)
Fzd2
Fzd3
Fzd4
Fzd5
Fzd6
Fzd7
Fzd8
Fzd9
Fzd10
Plasma membrane
biotin
Input
WT :
Tmem41bKO:
HA-FZD2
E-CAD
ACTIN
FZD2 enrichment at PM
ns
WT
Tmem41bKO

Figure S7

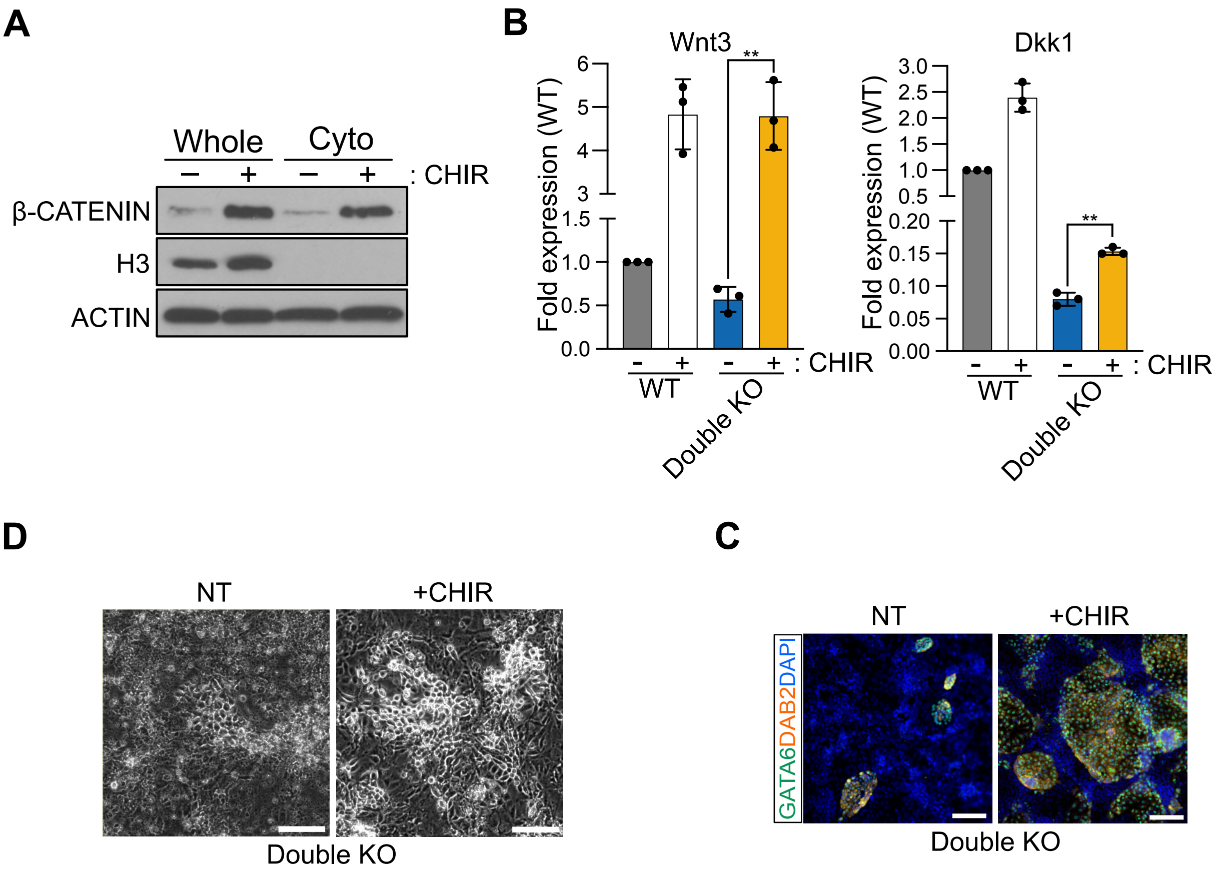
A
Whole
Cyto
–
+
–
+
: CHIR
β-CATENIN
H3
ACTIN
B
Wnt3
Dkk1
Fold expression (WT)
**
- + - + : CHIR
WT
Double KO
D
NT
+CHIR
Double KO
C
NT
+CHIR
GATA6DAB2DAPI
Double KO

Figure S8

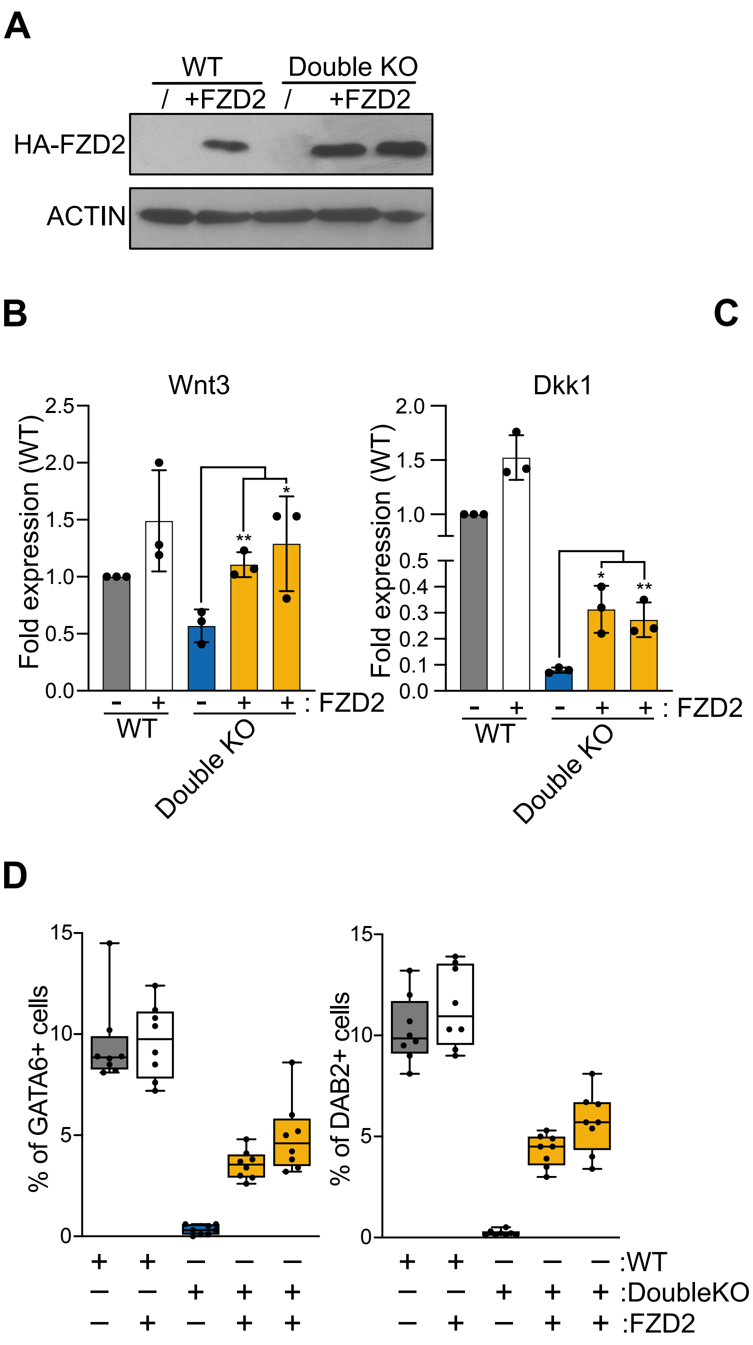

A
WT
Double KO
/ +FZD2 / +FZD2
HA-FZD2
ACTIN
B
Wnt3
Dkk1
Fold expression (WT)
- + - + + : FZD2
WT
Double KO
C
SOX17 GATA4 DAPI
GATA6 DAB2 DAPI
/
+FZD2
WT
Double KO
D
% of GATA6+ cells
% of DAB2+ cells
:WT
:DoubleKO
:FZD2

Figure S9

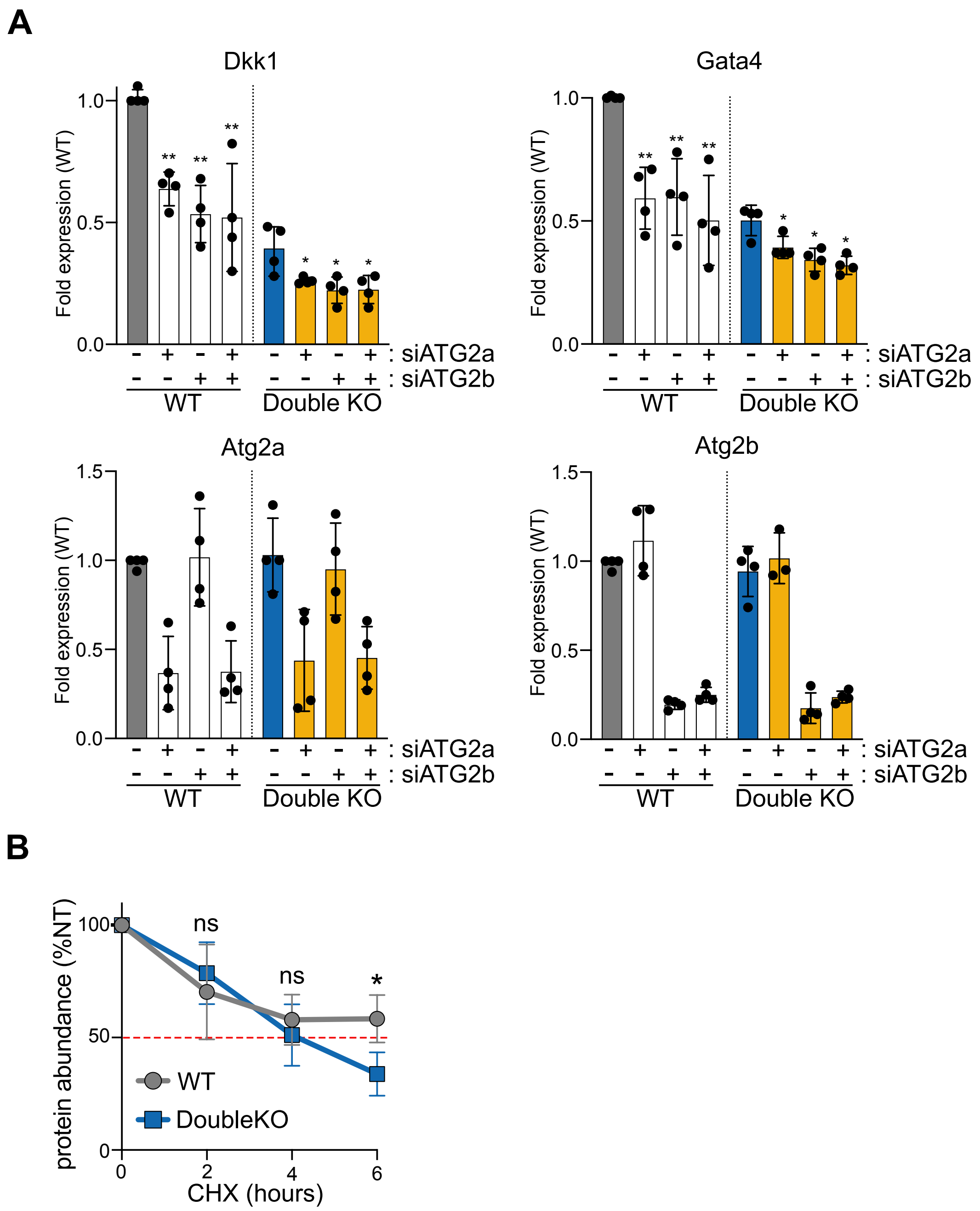
A
Dkk1
Gata4
Atg2a
Atg2b
Fold expression (WT)
1.5
1.0
0.5
0.0
**
*
: siATG2a
: siATG2b
WT
Double KO
B
protein abundance (%NT)
100
50
0
ns
ns
*
WT
DoubleKO
0
2
4
6
CHX (hours)

Figure S10

Figure 4D

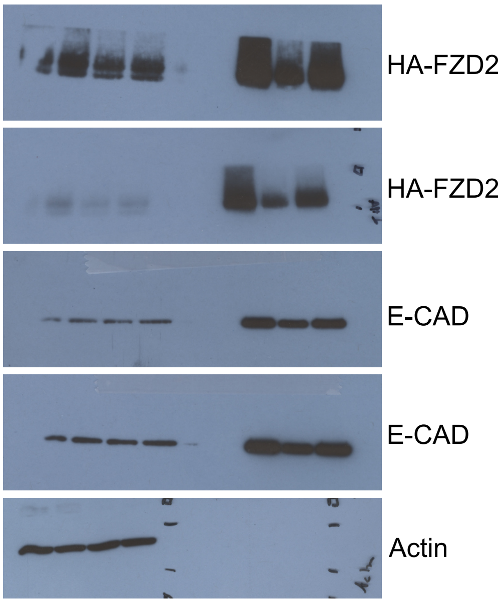

Figure S6E

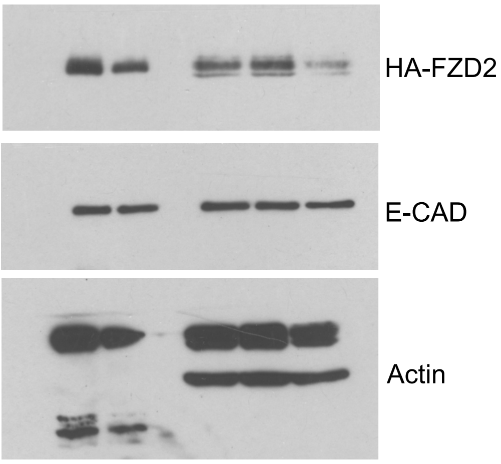

Figure S8A

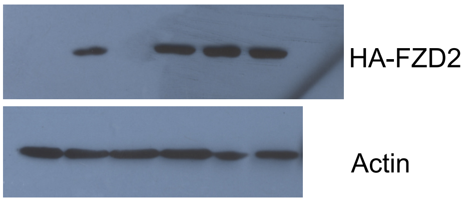

Figure 6B

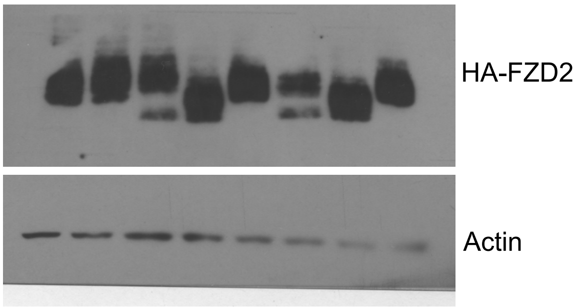

Figure 4F

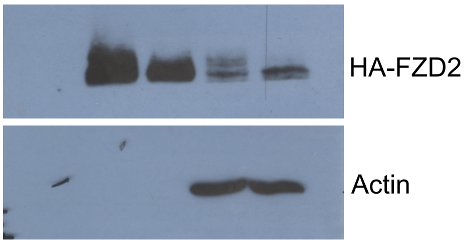

Figure 5A

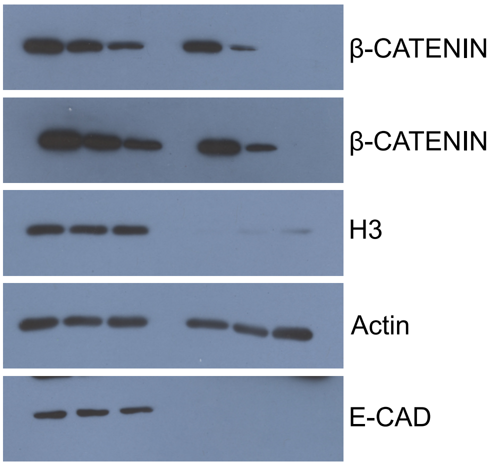

Figure S7A

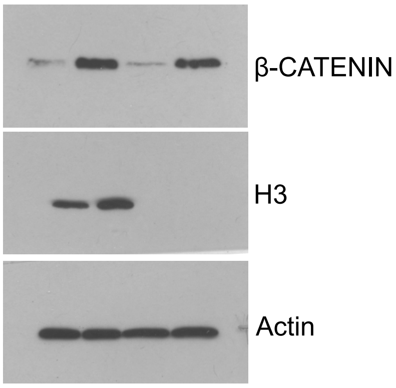

Figure 6F

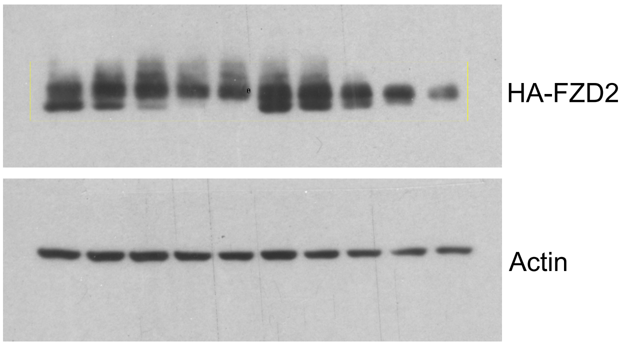

Supplement: Supplementary file 1 — Supplemental Information [file 41418_2024_1435_MOESM1_ESM.pdf]
